# Osteoarthritis and cardiovascular disease: A Mendelian randomization study

**DOI:** 10.3389/fcvm.2022.1025063

**Published:** 2022-11-18

**Authors:** Zhao Wang, Chan Kang, Pai Xu, Shuyi Zhang, Jae Hwang Song, Dongyang Wang, Shuai Yuan, Hyun Jong Lee, Meng Zhang, Zhihui Wang, Hao Sun, Ruobing Fan

**Affiliations:** ^1^Department of Orthopedic Surgery, Chungnam National University School of Medicine, Daejeon, South Korea; ^2^Department of Orthopedic Surgery, The Second Hospital of Fuzhou Affiliated to Xiamen University, Fuzhou, China; ^3^Department of Orthopedic Surgery, Konyang University Hospital, Daejeon, South Korea; ^4^School of Nursing, Binzhou Medical University, Yantai, China; ^5^Departmentof Surgery, School of Medicine, Ajou University, Suwon, South Korea; ^6^Affiliated Hospital of Chengde Medical University, Chengde, China; ^7^Graduate School of Hebei Medical University, Shijiazhuang, China

**Keywords:** Mendelian randomization (MR), Coronary heart disease (CHD), heart failure, stroke, knee osteoarthritis (KOA), hip osteoarthritis (HOA)

## Abstract

**Objective:**

This Mendelian randomization (MR) study aimed to investigate the causal relationship between osteoarthritis (OA) and cardiovascular disease (CVD).

**Methods:**

From a genome-wide association study of European ancestry, we selected single nucleotide polymorphisms for two types of OA, knee osteoarthritis (KOA) and hip osteoarthritis (HOA), as instrumental variables. We evaluated three types of CVD: coronary heart disease (CHD), heart failure (HF), and stroke. We used the traditional inverse variance weighting (IVW) method and other methods to estimate causality. Heterogeneity and sensitivity tests were also applied. Finally, we conducted a MR analysis in the opposite direction to investigate reverse causality.

**Results:**

IVW analysis showed that HOA significantly affected the incidence of HF [odds ratio (OR): 1.0675; 95% confidence interval (CI): 0.0182–0.1125, *P* = 0.0066]. HOA significantly affected the incidence of stroke (OR: 1.1368; 95% CI: 1.0739–1.2033, *P* = 9.9488e-06). CHD could dramatically affect the incidence of KOA (OR: 0.9011; 95% CI: 0.8442–0.9619, *P* = 0.0018). The rest of the results were negative.

**Conclusions:**

Our results revealed a potential causal relationship between HOA and risk of HF, and a potential causal relationship between HOA and risk of stroke. Our findings also suggested that CHD has a significant causal relationship with the risk of KOA. This paper may provide new ideas for the treatment of OA and CVD.

## Introduction

Cardiovascular disease (CVD) consists of diseases involving the heart or blood vessels, such as coronary heart disease (CHD), heart failure (HF), and stroke. CVD is the leading cause of morbidity and mortality in the general population worldwide. According to the World Health Organization, 17.8 million people die annually from CVD, which accounts for ~30% of all deaths worldwide (1). As a result, CVD places a considerable burden on individuals, families, and public finances. Some preventive measures can control the occurrence and development of CVD, so it is imperative to identify new cardiovascular risk factors and interventions.

Osteoarthritis (OA) is the most common joint disease in older patients, and it is the leading cause of joint dysfunction, disability, (2) and healthcare spending (3). With increasing population aging, obesity, and joint damage globally, the incidence of OA is rising, and studies show that 250 million people are being affected worldwide (4).

A meta-analysis of 15 articles, including a total of 358,944 participants (80,911 patients with OA and 29,213 with CVD), identified OA as an essential risk factor for CVD (5). Another study in from Rotterdam showed that disability predicts CVD (6). The association between OA and CVD remains unknown. At the same time, Most of the previous studies came from observational studies and cannot be used to determine causality due to the possibility of confounding and reverse causality (7). So we aimed to clarify this relationship with a Mendelian randomization (MR) analysis.

An MR analysis uses genetic variation associated with a specific exposure of interest to study the causal impact of modifying exposure (i.e., potential risk factors) on health, social, and economic outcomes (8). It can minimize confounding factors and reverse causality problems found in traditional observational epidemiological studies. To avoid bias and improve the reliability of the results, MR is widely used to evaluate the relationship between risk factors and disease prognosis (9). This analysis is used for estimating the causal effect of exposure on outcomes using single nucleotide polymorphisms (SNPs) associated with exposure-related genes as instrumental variables under specific assumptions (10). Two-sample MR analysis requires summary-level data from two independent genome-wide association studies (GWAS) to identify putative exposures and outcomes. Eliminating confounders improves the reliability of results from MR analysis (11). To the best of our knowledge, this is the first two-sample MR study to explore the causal effect between CVD and OA.

## Methods

### Study overview

**Figure 1:**
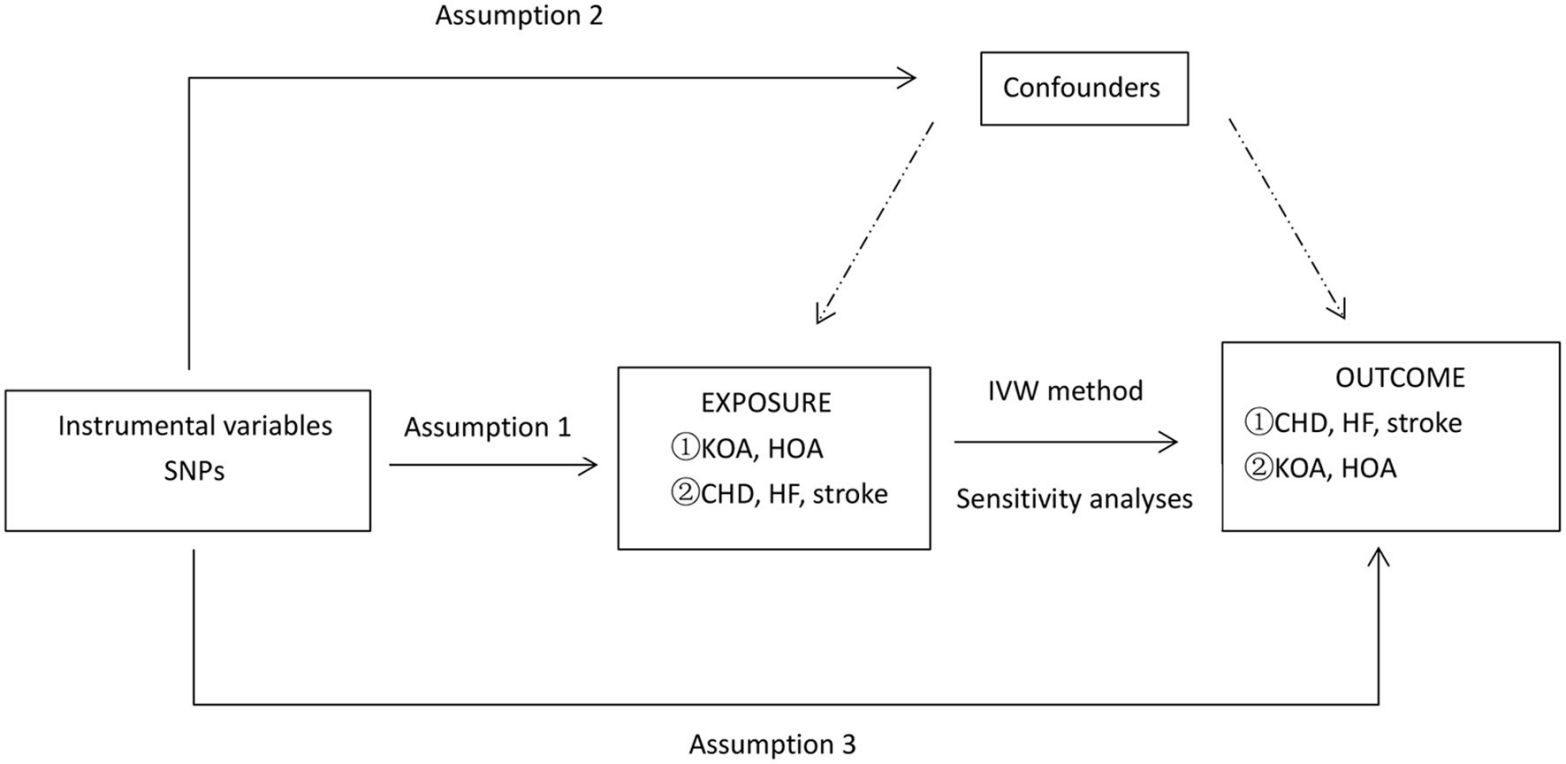
An overview of the study design. SNP, single-nucleotide polymorphism; CHD, coronary heart disease; HF, heart failure; KOA, knee osteoarthritis; HOA, hip osteoarthritis; IVW, inverse-variance weighted; IV: instrumental variable. Symbol ① represents a forward Mendelian analysis process with osteoarthritis as the exposure and cardiovascular disease as the outcome. Symbol ② defines a reverse Mendelian analysis process with cardiovascular disease as the exposure and osteoarthritis as the outcome. Assumption 1, the genetic variants selected as IVs should be strongly associated with the risk factor of interest. Assumption 2, the genetic variants used as IVs should not be associated with confounders. Assumption 3, the IVs should affect the risk of the outcome merely through the risk factor, not any alternative pathways. *Sensitivity analyses: MR-PRESSO and leave-one-out test.

In this MR analysis, we selected the two most common types of osteoarthritis, knee osteoarthritis (KOA) and hip osteoarthritis (HOA), as the study subjects. To represent CVDs, the more common CHD, HF, and stroke were selected. In the forward MR method analysis, we used SNPs closely associated with CHD, HF, and stroke as instrumental variables. In the reverse MR method analysis, we used SNPs closely related to KOA and HOA as instrumental variables. An overview of the study design is shown in Figure 1.

### GWAS summary statistics for KOA and HOA

KOA and HOA data (sample sizes of 393,873 and 403,124, respectively) were derived from the Integrative Epidemiology Unit (IEU) GWAS database (https://gwas.mrcieu.ac.uk). The raw data could be found in the study by Tachmazidou et al. (12). All data were from individuals of European descent (Supplementary Table H1).

### GWAS summary statistics for CHD, HF and stroke

Data on CHD, HF, and stroke were all obtained from the Integrative Epidemiology Unit (IEU) GWAS database (https://gwas.mrcieu.ac.uk). The data were collected only from Europeans (Supplementary Table H1). We used data from 22,233 patients with CHD matched with 64,762 healthy control patients [raw data are available in the study by Schunkert et al. (13)], 47,309 patients with HF matched with 930,014 controls [raw data can be found in the study by Malik R et al. (14)], and 34,217 patients with stroke matched with 406,111 controls [raw data are available in the study by Shah S et al. (15)].

### Genetic tool variables

We set uniform filtering criteria for the tool variables. *P* < 5 × 10^−8^ indicated statistical significance. Linkage disequilibrium analysis of the corresponding SNPs was performed for each instrumental variable. To eliminate the effect of known confounders on the causality estimates, SNPs associated with CVD and OA were manually removed using PhenoScanner (http://www.phenoscanner.medschl.cam.ac.uk). Potential confounders associated with OA included body mass index, systolic blood pressure, low-density lipoprotein cholesterol, smoking, and education (16). Potential confounders for CVD include common risk factors for CVD such as hypertension.

According to the hypothesis of the MR analysis, the selected instrument SNP should be closely related to the exposure. To test whether there is a weak instrumental variable bias, we subsequently calculated the F statistic using the following formula: *F* = [R2combined/(1-R 2combined)] × [(N-K-1)/K], where *N* = GWAS sample size, *K* = number variants comprising the instrument. R2 was calculated using the following formula: R2 = [beta.exposure2]/[se.exposure2 ×N + beta.exposure2]; R2 combined = SUM[R2], where beta.exposure = SNP exposure effect and se. exposure = standard error of SNP exposure effect. If the F statistic of the instrument exposure association is much greater than 10, a weak instrument variable bias is unlikely (17).

### Statistical analysis

We assessed the causal relationship of OA (HOA and KOA) on CVD (CHD, HF and stroke), and then to test the causal relationship of CVD (HOA and KOA) on OA (HOA and KOA), we conducted a reverse MR study. This is the bidirectional MR study.

We used the inverse-variance weighted (IVW) analysis as the primary analysis, (18) and the Weighted median and MR-Egger as other analysis methods (19, 20). We used the Cochran'sQ analysis to assess the heterogeneity. *P*-values > 0.05 indicate no heterogeneity (21). At this time, the fixed-effect IVW method is considered the primary method (22). For the sensitivity analyses, we used a leave-one-out test and, after removal, revisited the MR analysis to check if there were variables affecting the causal effect estimates (18). The horizontal pleiotropy test was also applied in the experiments to measure the average pleiotropy between the tool variables by the intercept term of the MR-Egger (20). The presence of pleiotropy was also assessed by Mendelian Randomization Pleiotropy RESidual Sum and Outlier (MR-PRESSO), and effect estimates were reassessed after exclusion of outliers (23). This study was performed using the R package of “TwoSampleMR” and “MR-PRESSO” (23, 24). All statistical data analyses were performed using R software version 4.1.3.

### Ethics

Our analysis used either published studies or publicly available GWAS summary data. Raw data were not collected for the analysis; therefore, ethics committee approval was not required. Each study included was approved by their institutional ethics review board, and all participants provided written informed consent.

## Results

### Selection of the tool variables

The SNPs associated with the confounders were removed (Supplementary Table H2). Details used for the SNP related to CVD (CHD, HF, and stroke) and the SNP associated with OA (KOA and HOA) are listed in Supplementary Tables D1–12. In our study, the statistics for each exposure association were much >10, indicating a small probability of weak instrument variable bias (Table 1).

**Table 1:** Mendelian randomization estimates of KOA and HOA on CHD, HF, and stroke.

**Exposure**	**Outcomes**	**IVW**					**Weighted median**			**MR-Egger**				***F*** **-statistic**
		**Odds ratio**	**95% CI**	***P*** **-value**	***Q-*** **value**	**Heterogeneity** ***P*-value**	**Odds ratio**	**95% CI**	**P value**	**Odds ratio**	**95% CI**	***P*** **-value**	**Pleiotropy** ***P*-value**	
KOA	CHD	0.9607	0.5994, 1.5399	0.8679	0.2880	0.0827	1.0253	0.6564, 1.6017	0.9124	39.6918	0.7579, 2078.8180	0.3195	0.3157	38.1196
	HF	0.9330	0.8017, 1.0857	0.3697	10.4018	0.0646	0.8964	0.7758, 1.0358	0.1424	0.6324	0.3054, 1.3093	0.2847	0.3448	39.1124
	Stroke	0.9347	0.7907, 1.1049	0.4290	6.1001	0.2965	0.8796	0.7246, 1.0676	0.1942	0.2968	0.0203, 4.3307	0.4246	0.4479	36.3175
HOA	CHD	0.9688	0.8621, 1.0887	0.5942	4.4116	0.7313	0.9790	0.8475, 1.1308	0.7727	1.1482	0.7492, 1.7596	0.5492	0.4484	57.5553
	HF	1.0675	1.0184,1.1191	0.0066	12.5076	0.7084	1.0471	0.9809,1.1177	0.1672	1.0005	0.8310,1.2047	0.9956	0.4901	47.5058
	Stroke	1.1368	1.0739,1.2033	9.9488e-06	17.4521	0.4922	1.1374	1.0527,1.2290	0.0011	1.0897	0.8841,1.3432	0.4319	0.6854	47.3617

CHD, coronary heart disease; HF, heart failure; KOA, knee osteoarthritis; HOA, hip osteoarthritis; IVW, inverse-variance weighted; CI, confidence interval; MR Mendelian randomization.

### Causation and effect of exposure (KOA, HOA) for outcome (CHD, HF, stroke)

We evaluated the causality of exposure (KOA and HOA) on outcome (CHD, HF, and stroke) in this MR analysis (Table 1). HOA had a significant effect on the incidence of HF and stroke, but not CHD. KOA was also not significantly associated with HF, stroke, and CHD.

#### Effect of Exposure (KOA) on Outcome (CHD, HF, stroke)

KOA showed no remarkable influence on CVD (CHD, HF, or stroke) based on the IVW, weighted median, and MR-Egger analyses (Table 2). The estimated effect sizes of exposure (KOA) on outcome (CHD, HF, and stroke) are shown in the scatter plot (Supplementary Figures R1–3). The funnel plot provides a simple method for detecting directional-level pleiotropic tests, as shown in Supplementary Figures S1–3. The forest plot reflects the results estimated by a single SNP using the Wald ratio method and is shown in the Supplementary Figures T1–3. We found no significant heterogeneity (*P* > 0.05) and no substantial evidence of horizontal pleiotropy from the MR-Egger intercept (intercept *P* > 0.05).

**Table 2:** Mendelian randomization estimates of CHD, HF, and stroke on KOA and HOA.

**Exposure**	**Outcomes**	**IVW**					**Weighted median**			**MR-Egger**				***F*** **-statistic**
		**Odds ratio**	**95% CI**	***P*** **-value**	***Q-*** **value**	**Heterogeneity** ***P*-value**	**Odds ratio**	**95% CI**	**P value**	**Odds ratio**	**95% CI**	***P*** **-value**	**Pleiotropy** ***P*-value**	
CHD	KOA	0.9011	0.8442, 0.9619	0.0018	12.1158	0.1461	0.9126	0.8498, 0.9800	0.0118	0.8949	0.7375, 1.0858	0.2974	0.9420	49.5032
HF		1.2225	0.9861, 1.5158	0.0669	4.3909	0.2222	1.1418	0.9137, 1.427	0.2436	1.0306	0.5458, 1.9461	0.9344	0.6260	45.3623
Stroke		1.0127	0.8964, 1.1441	0.8388	4.0983	0.5353	0.9800	0.8394, 1.1441	0.7981	1.1352	0.4966, 2.5949	0.7787	0.7979	38.4571
CHD	HOA	0.9968	0.9191, 1.081	0.9377	15.3800	0.9962	0.9983	0.9156, 1.0885	0.9692	0.9654	0.7598, 1.2268	0.7818	0.9962	49.5032
HF		1.0412	0.8661, 1.2517	0.6677	5.1707	0.7052	1.1162	0.9117, 1.4822	0.2247	1.1607	0.6618, 2.0357	0.3956	5.1689	43.6276
Stroke		1.1064	0.9100, 1.3452	0.3103	11.0678	0.0863	1.0977	0.8948, 1.3466	0.3714	2.7712	0.7927, 9.6876	0.1713	0.2060	37.5227

CHD, coronary heart disease; HF, heart failure; KOA, knee osteoarthritis; HOA, hip osteoarthritis; IVW, inverse-variance weighted; CI, confidence interval; MR Mendelian randomization; OR, odds ratio.

#### Effect of exposure (HOA) on outcome (CHD, HF, stroke)

**Figure 2:**
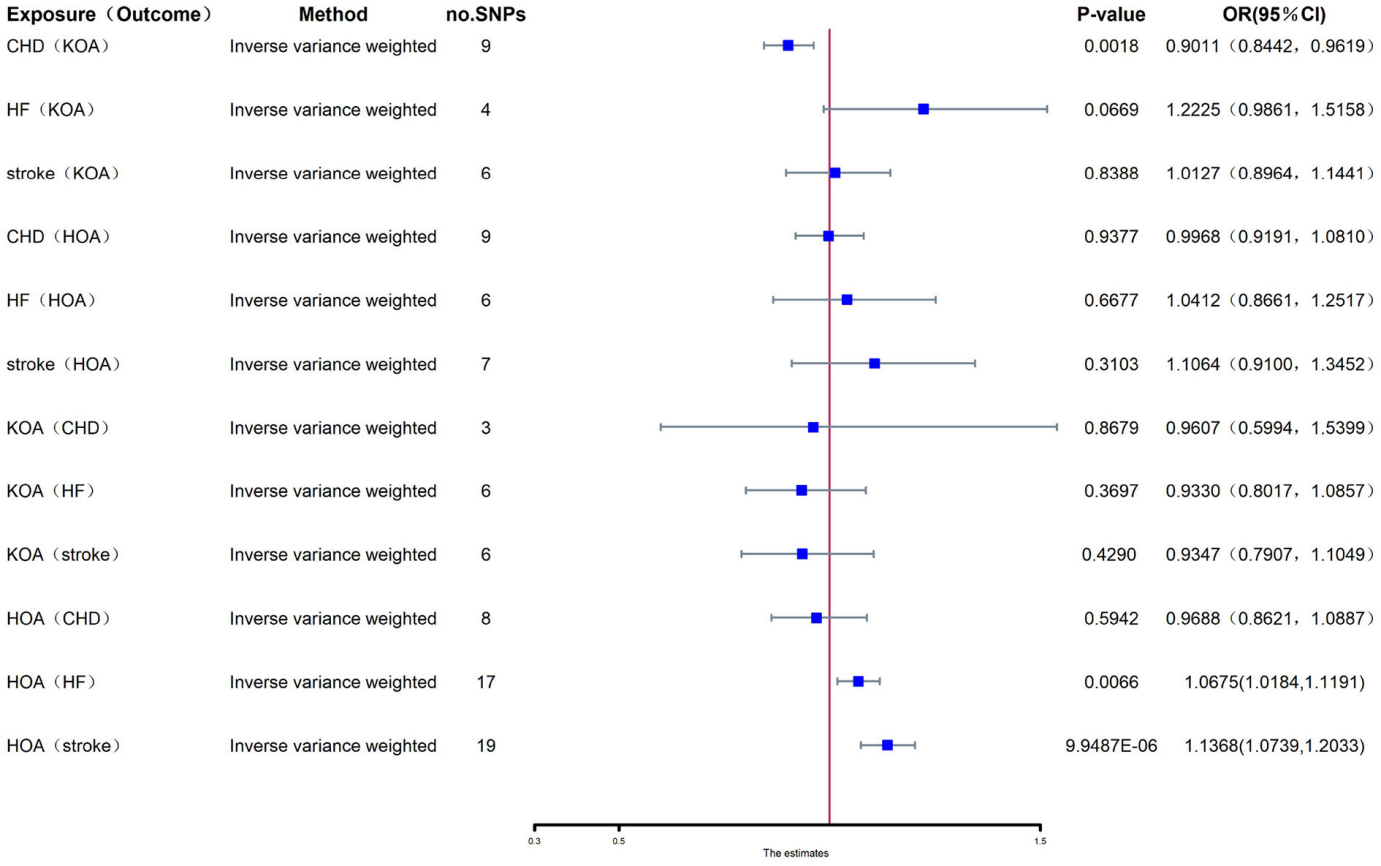
CHD, coronary heart disease; HF, heart failure; KOA, knee osteoarthritis; HOA, hip osteoarthritis; CI, confidence interval; no.SNPs, number of single nucleotide polymorphisms.

HOA can significantly affect the incidence of HF, and each increasing standard deviation in the risk of HOA had a 6.75% higher risk of HF in the IVW analysis [odds ratio (OR): 1.0675; 95% confidence interval (CI): 0.0182–0.1125, *P* = 0.0066] (Figure 2). The weighted median method and the MR-Egger method showed negative results (Table 1). HOA had a significant effect on the incidence of stroke, and each increased standard deviation in the risk of HOA had an increased risk of stroke by 13.68% in the IVW analysis (OR: 1.1368; 95% CI: 1.0739–1.2033, *P* = 9.9488e-06) (Figure 2). The weighted median approach also confirmed this positive finding, and the MR-Egger method showed negative results (Table 1). HOA showed no influence on CHD based on the IVW, weighted median, and MR-Egger analyses (Table 2). The estimated effect sizes for exposure (HOA) and outcome (CHD, HF, and stroke) are shown in the scatter plot (Supplementary Figures R4–6). The funnel diagram is shown in Supplementary Figures S4–6. The forest plot is shown in Supplementary Figures T4–6. We found no significant heterogeneity (*P* > 0.05). Our analysis showed no substantial evidence of horizontal pleiotropy from the MR-Egger intercept (intercept *P* > 0.05).

### The causal effect of exposure (CHD, HF, stroke) on outcome (KOA, HOA)

We also evaluated the causality of exposure (CHD, HF, and stroke) on the outcome (KOA and HOA) in the MR analysis (Table 2). In the reverse MR analysis, CHD significantly affected KOA. However, CHD had no relationship with the incidence of HOA. No special relationship was found between exposure (HF and stroke) and outcome (KOA and HOA).

#### Effects of exposure (CHD, HF, stroke) on outcome (KOA)

CHD could dramatically affect the incidence of KOA. The IVW analysis showed that with every standard deviation increase in the risk of CHD, the risk of having KOA was reduced by 9.89% (OR: 0.9011; 95% CI: 0.8442–0.9619, *P* = 0.0018) (Figure 2). The weighted median approach also confirmed this positive finding. However, HF and stroke showed no influence on KOA based on IVW, weighted median, and MR-Egger analyses (Table 2). The estimated effect sizes of exposure (CHD, HF, and stroke) on the outcome (KOA) are shown in the scatter plot (Supplementary Figures R7–9). The funnel diagram is shown in Supplementary Figures S7–9. The forest plot is shown in Supplementary Figures T7–9. We found no significant heterogeneity in the heterogeneity testing (*P* > 0.05). Our analysis showed no substantial evidence of horizontal pleiotropy from the MR-Egger intercept (*P* > 0.05).

#### Effect of exposure (CHD, HF, stroke) on outcome (HOA)

CHD, HF, and stroke showed no influence on HOA based on the IVW, weighted median, and MR-Egger analyses (Table 2). The estimated effect sizes of exposure (CHD, HF, and stroke) on the outcome (HOA) are shown in the scatter plot (Supplementary Figures R10–12). The funnel diagram is shown in Supplementary Figures S10–12. The forest plot is shown in Supplementary Figures T10–12. We found no significant heterogeneity (*P* > 0.05). The MR-Egger intercept showed no substantial evidence of horizontal pleiotropy (intercept *P* > 0.05).

### Evaluation and sensitivity analysis of the assumptions

We used MR-PRESSO analysis to test for horizontal pleiotropy. In the above studies, MR-PRESSO analysis showed that several abnormal SNPs were found in the instrumental variables for the specific associations of stroke on KOA and HOA on stroke (Supplementary Table E1). When we removed these outliers, no MR-PRESSO outliers' exceptions were found (Supplementary Table F1).

Considering the impact of individual SNPs, we performed a leave-one-out method for sensitivity analysis. In the above studies, the leave-one-out method showed that several abnormal SNPs were found in the instrumental variables for the specific association of stroke with HOA and HOA with HF (Supplementary Table E2). When these outliers were removed, we found no leave-one-out method outliers (Supplementary Figures H1−12). This result suggests that the causal relationships were not reliant on any single SNP.

## Discussion

This is the most comprehensive MR study evaluating the association between OA and CVD risk. Our MR analysis results suggest that patients with CHD have a reduced risk of KOA. Additionally, patients with HOA have an increased incidence of HF and stroke. Furthermore, this association was robust in the sensitivity analysis, and none of the instrumental variables severely affected the outcome variable.

The mechanisms underlying the observed association between OA and CVD risk are unknown; however, several factors may explain this relationship. First, the two diseases share a few common risk factors. Epidemiological studies have provided evidence of an association between OA and most traditional cardiovascular risk factors, including obesity, diabetes mellitus, and hyperlipemia (25). Second, patients with CHD typically reduce the amount of exercise, which directly reduces weight bearing of the knee joint, resulting in a reduction in the incidence of KOA (26). At the same time, drugs for CHD can improve KOA by regulating ion channels (27). Third, chronic pain can increase the risk of stroke. This mechanism is associated with the stress response and could negatively impact the vasculature by the hypothalamic-pituitary-adrenal axis (28, 29). The most commonly prescribed medications for pain relief in patients with OA are non-steroidal anti-inflammatory drugs, which are associated with an increased risk of vascular events (30). OA and cerebrovascular disease are also not directly related, but their association can be explained by physical activity, depression, and sleep disorders (31). Finally, between HOA and HF, the risk of HF is increased due to the reduced amount of exercise in patients with HOA (32). Studies have shown a connection between zinc ions and the development of OA, (33) and elevated zinc ions can also lead to an increased risk of HF (34).

This study has several limitations. First, the results of other MR methods (MR Egger, weighted median, simple and weighted mode) were not entirely consistent with the IVW method in the univariate MR analysis. However, according to the principle of method selection, in the absence of heterogeneity and pleiotropy, the estimated results of IVW can be preferentially used. Second, the exposure and outcome studies used in the two-sample MR analyses should not involve overlapping participants. We could not estimate the extent of the overlap in this study. However, using powerful tools can minimize the bias in sample overlap (e.g., an F statistic of much larger than 10) (35). Third, MR studies revealed the potential causal effects of CHD on KOA and the impact of HOA on HF and stroke. Still, other outcomes did not correlate, and the factors causing this inconsistency remain elusive. Fourth, due to the limitations of the GWAS summary statistics, the MR analysis based on different ages, gender, and height was not feasible. Fifth, our MR analysis was limited to populations of European ancestry, and the conclusions may not necessarily apply to populations of Asian ancestry. Sixth, even if confounding has been removed in this study, the influence of third-party conditions cannot be excluded, so the results may be non-linear and need further confirmation.

Because CVD and OA are widespread diseases, it is crucial to find the link between OA and CVD from a public health perspective. In the general middle-aged population, screening for HOA status and traditional cardiovascular risk factors may be considered for early intervention to reduce future HF and stroke events. Patients with HOA should be aware of the risk of HF and stroke. Among clinicians, cardiovascular risk must be considered when prescribing any non-steroidal anti-inflammatory drugs to patients with HOA. Clinicians should also focus on managing pain in patients with HOA, particularly those with HF and stroke. Reducing the risk of HF and stroke can reduce some of the mortality and economic burden on society. Further studies on the relationship between CHD and KOA could use CHD drugs to identify more effective medicines for prevention and treatment of KOA. This method of researching drugs will provide new perspectives on the treatment of KOA.

## Conclusion

In conclusion, this MR study provides strong evidence that HOA is a significant risk factor for HF and stroke. Patients with CHD have a reduced risk of KOA. Given the high prevalence and incidence of OA and CVD in the general population, this study, of the relationship between OA and CVD, has important dual clinical and public health implications.

## Data availability statement

Publicly available datasets can be found here: the Integrative Epidemiology Unit (IEU) GWAS database (https://gwas.mrcieu.ac.uk).

## Author contributions

ZhaW and CK designed the study. ZhaW, PX, and SZ performed data analysis and drafted the manuscript. JS, CK, PX, SZ, and DW revised the manuscript. MZ, ZhiW, and HS collected the data. SY, HL, and RF provided the resources. CK lead the study. All authors contributed to the article and approved the submitted version.

## Conflict of interest

The authors declare that the research was conducted in the absence of any commercial or financial relationships that could be construed as a potential conflict of interest.

## Publisher's note

All claims expressed in this article are solely those of the authors and do not necessarily represent those of their affiliated organizations, or those of the publisher, the editors and the reviewers. Any product that may be evaluated in this article, or claim that may be made by its manufacturer, is not guaranteed or endorsed by the publisher.
