# Supplementary figures and images for: Osteoarthritis and cardiovascular disease: A Mendelian randomization study
Source: Front Cardiovasc Med. 2022 Nov 18;9:1025063. doi: 10.3389/fcvm.2022.1025063 (PMC9717609; doi:10.3389/fcvm.2022.1025063)

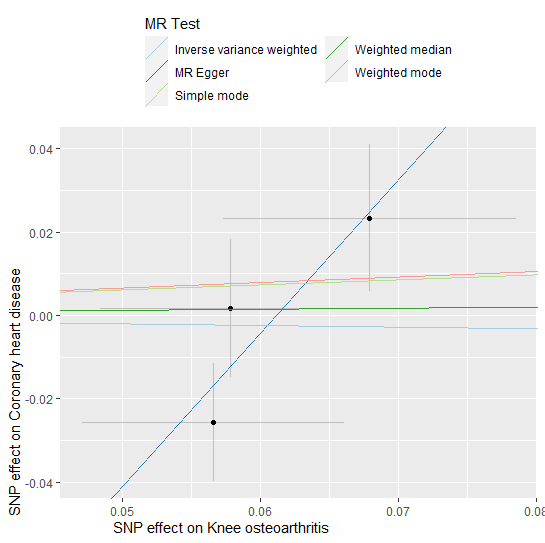

Supplement: Supplementary file 1 [file Data_Sheet_1.ZIP › Additional file 1 Scatter plot figure/R1 KOA on CHD.png]

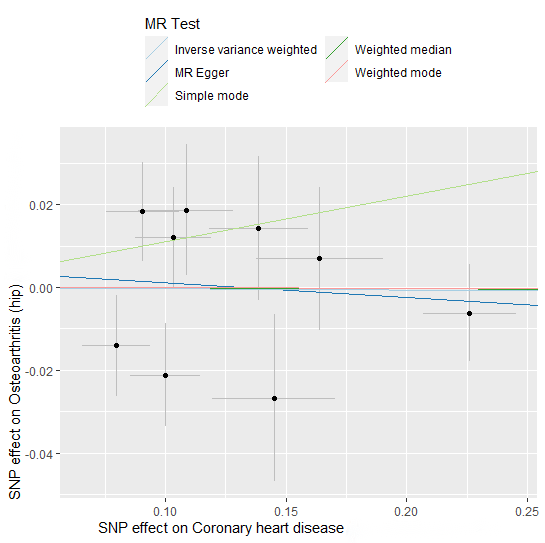

Supplement: Supplementary file 1 [file Data_Sheet_1.ZIP › Additional file 1 Scatter plot figure/R10 CHD on HOA.png]

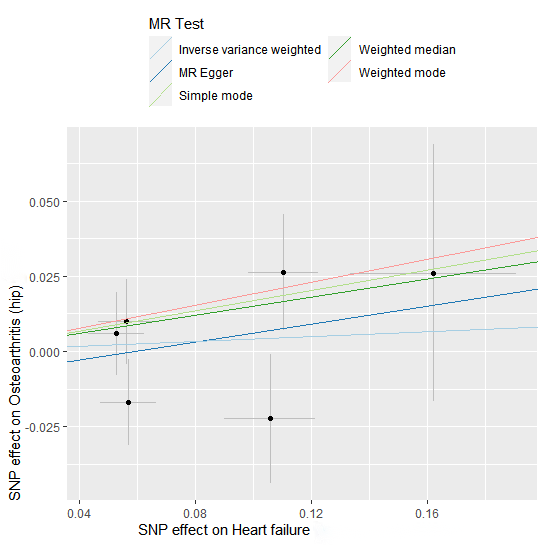

Supplement: Supplementary file 1 [file Data_Sheet_1.ZIP › Additional file 1 Scatter plot figure/R11 HF to HOA.png]

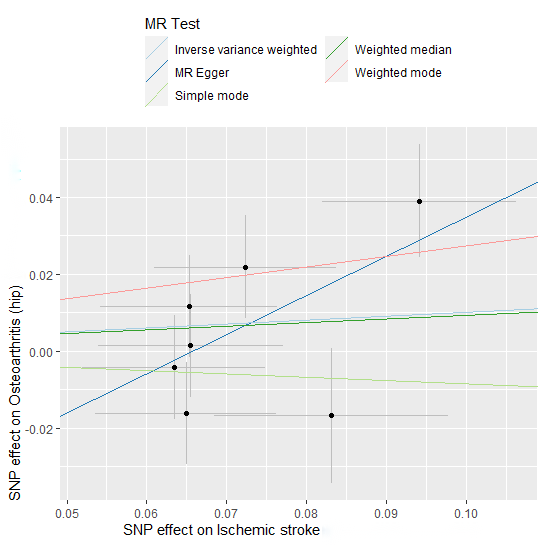

Supplement: Supplementary file 1 [file Data_Sheet_1.ZIP › Additional file 1 Scatter plot figure/R12 Stroke to HOA.png]

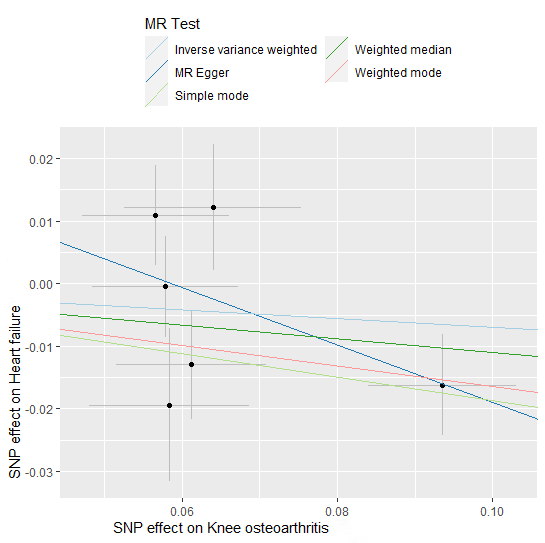

Supplement: Supplementary file 1 [file Data_Sheet_1.ZIP › Additional file 1 Scatter plot figure/R2 KOA on HF.png]

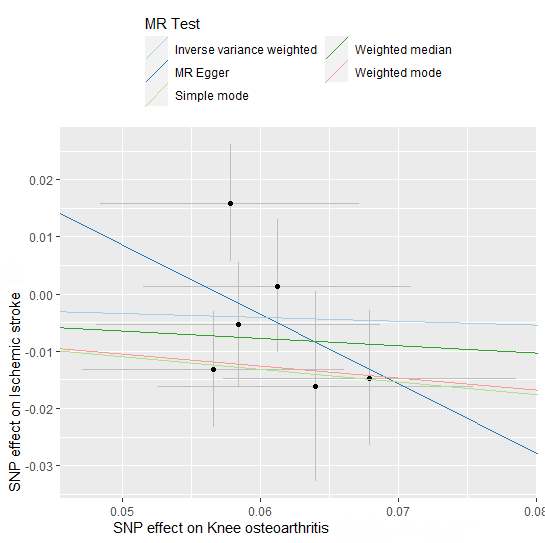

Supplement: Supplementary file 1 [file Data_Sheet_1.ZIP › Additional file 1 Scatter plot figure/R3 KOA on Stroke.png]

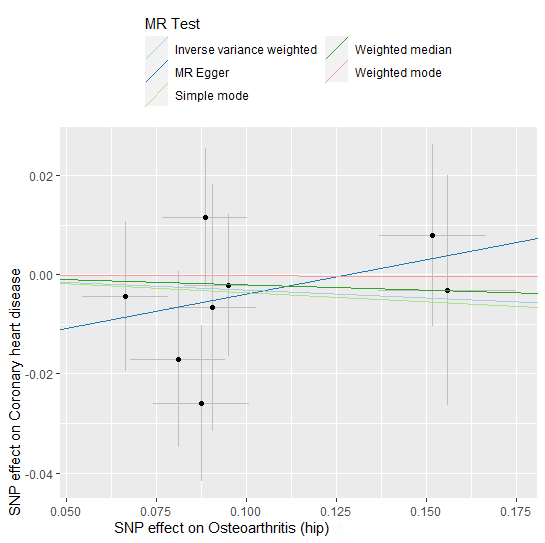

Supplement: Supplementary file 1 [file Data_Sheet_1.ZIP › Additional file 1 Scatter plot figure/R4 HOA on CHD.png]

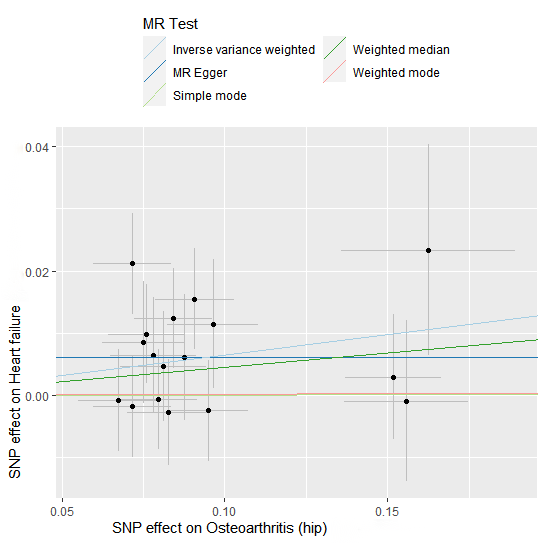

Supplement: Supplementary file 1 [file Data_Sheet_1.ZIP › Additional file 1 Scatter plot figure/R5 HOA on HF.png]

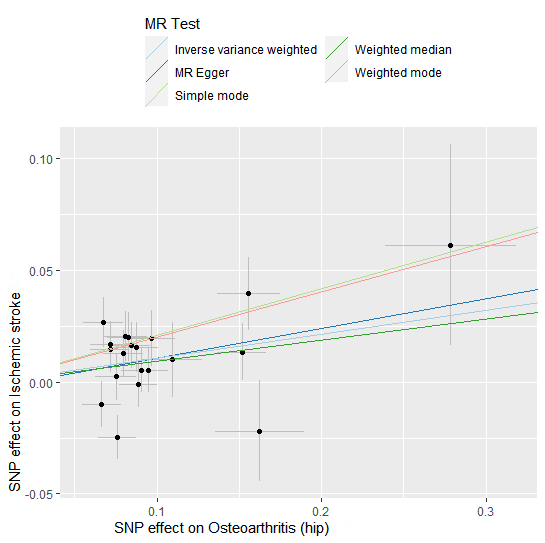

Supplement: Supplementary file 1 [file Data_Sheet_1.ZIP › Additional file 1 Scatter plot figure/R6 HOA on Stroke.png]

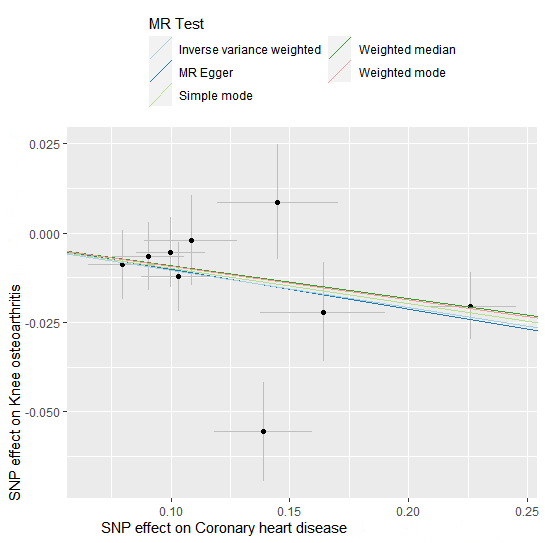

Supplement: Supplementary file 1 [file Data_Sheet_1.ZIP › Additional file 1 Scatter plot figure/R7 CHD on KOA.png]

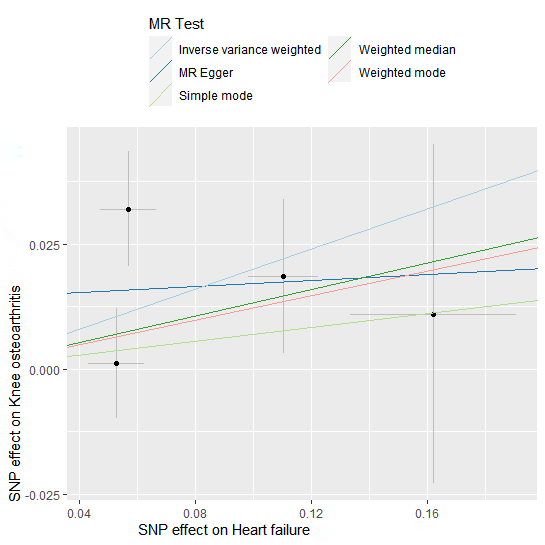

Supplement: Supplementary file 1 [file Data_Sheet_1.ZIP › Additional file 1 Scatter plot figure/R8 HF on KOA.png]

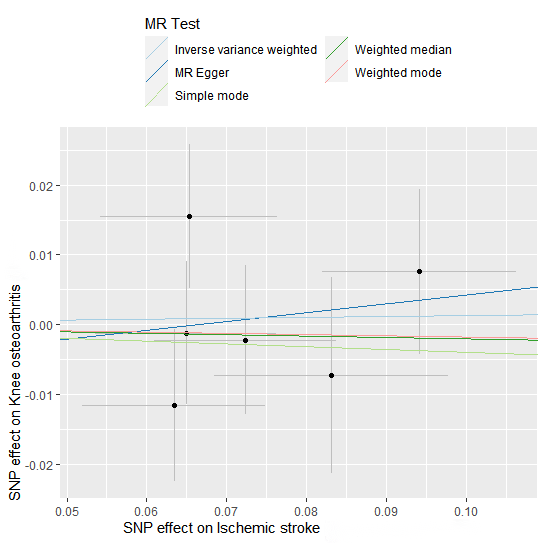

Supplement: Supplementary file 1 [file Data_Sheet_1.ZIP › Additional file 1 Scatter plot figure/R9 Stroke on KOA.png]

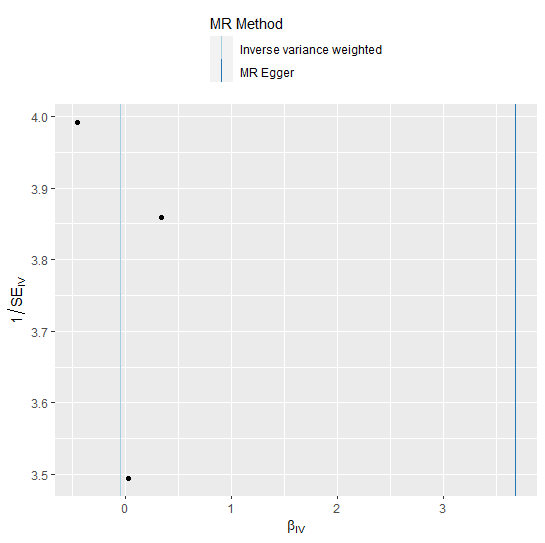

Supplement: Supplementary file 1 [file Data_Sheet_1.ZIP › Additional file 2 Funnel plot figure/S1 KOA on CHD.png]

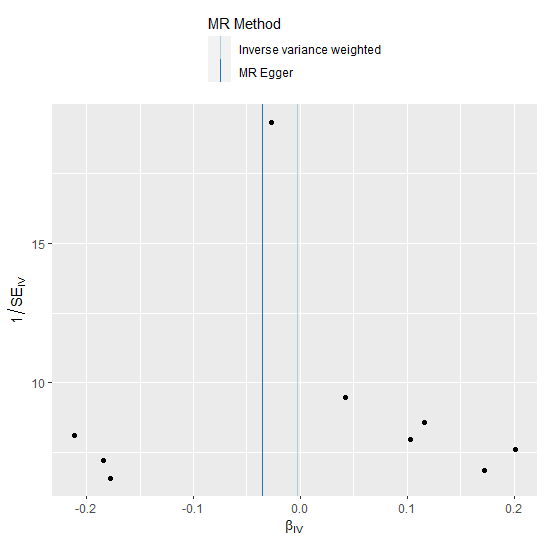

Supplement: Supplementary file 1 [file Data_Sheet_1.ZIP › Additional file 2 Funnel plot figure/S10 CHD on HOA.png]

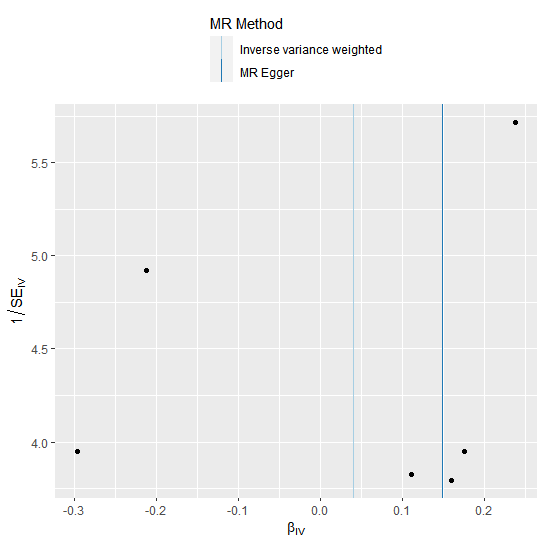

Supplement: Supplementary file 1 [file Data_Sheet_1.ZIP › Additional file 2 Funnel plot figure/S11 HF on HOA.png]

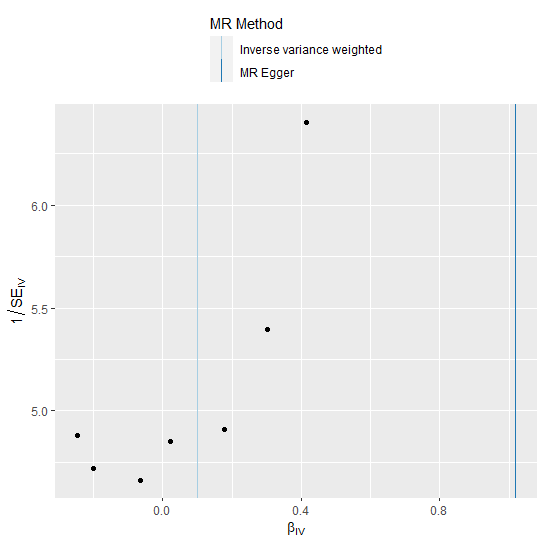

Supplement: Supplementary file 1 [file Data_Sheet_1.ZIP › Additional file 2 Funnel plot figure/S12 Stroke on HOA.png]

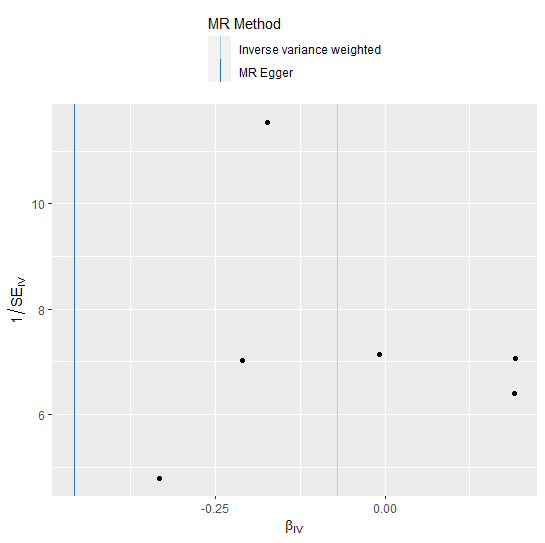

Supplement: Supplementary file 1 [file Data_Sheet_1.ZIP › Additional file 2 Funnel plot figure/S2 KOA on HF.png]

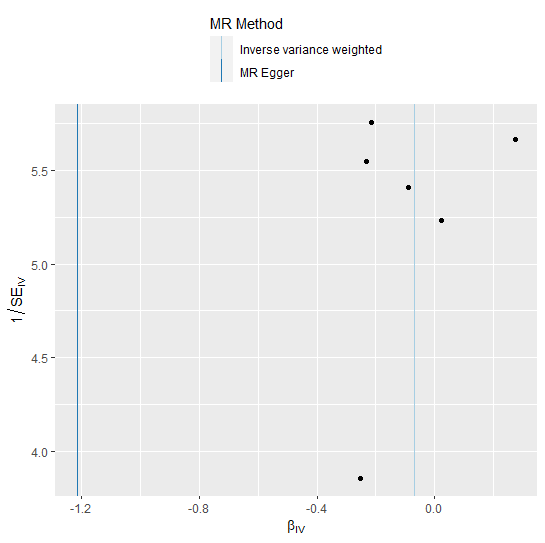

Supplement: Supplementary file 1 [file Data_Sheet_1.ZIP › Additional file 2 Funnel plot figure/S3 KOA on Stroke.png]

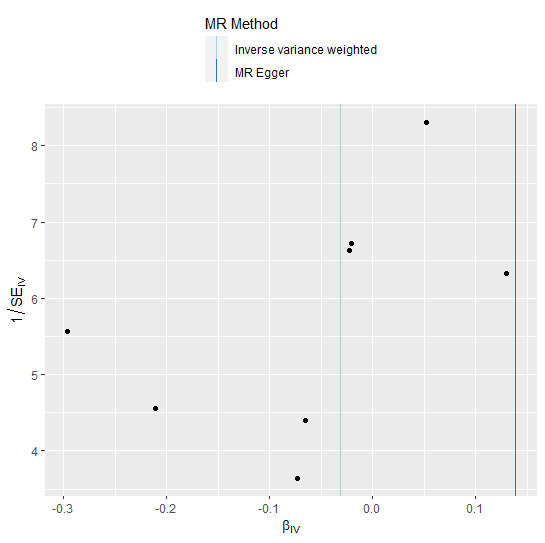

Supplement: Supplementary file 1 [file Data_Sheet_1.ZIP › Additional file 2 Funnel plot figure/S4 HOA on CHD.png]

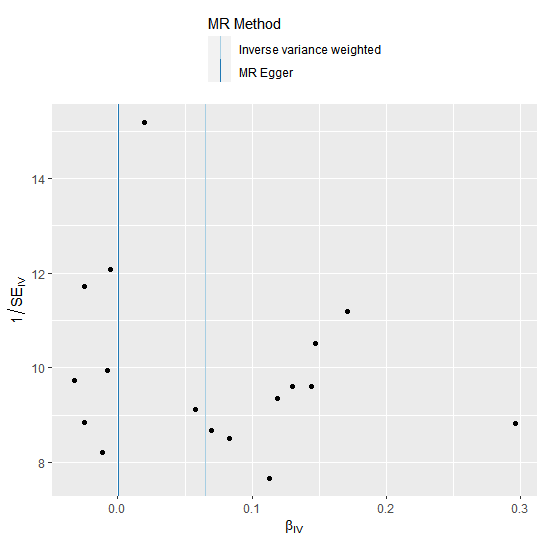

Supplement: Supplementary file 1 [file Data_Sheet_1.ZIP › Additional file 2 Funnel plot figure/S5 HOA on HF.png]

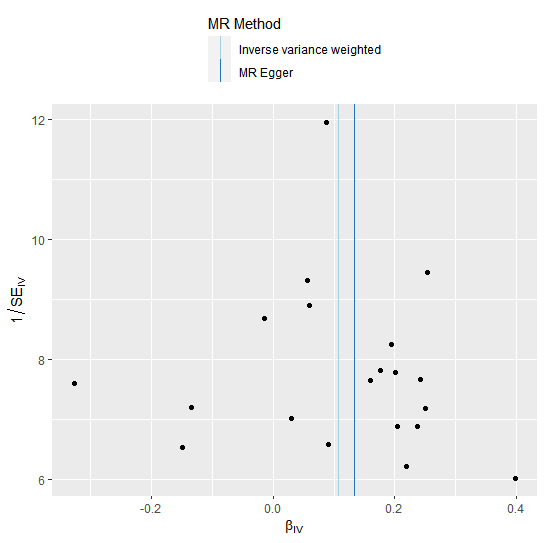

Supplement: Supplementary file 1 [file Data_Sheet_1.ZIP › Additional file 2 Funnel plot figure/S6 HOA on Stroke.png]

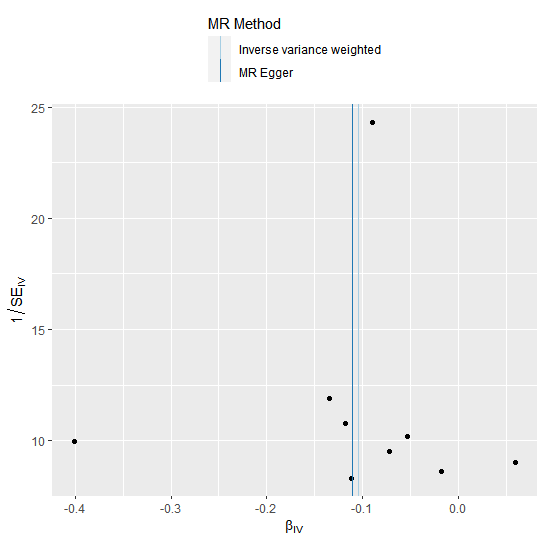

Supplement: Supplementary file 1 [file Data_Sheet_1.ZIP › Additional file 2 Funnel plot figure/S7 CHD on KOA.png]

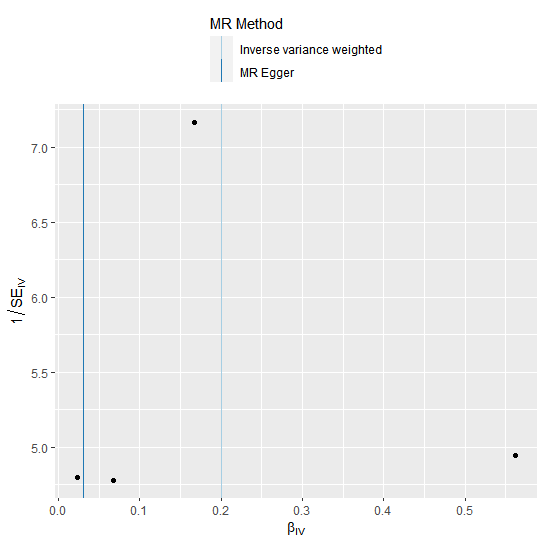

Supplement: Supplementary file 1 [file Data_Sheet_1.ZIP › Additional file 2 Funnel plot figure/S8 HF on KOA.png]

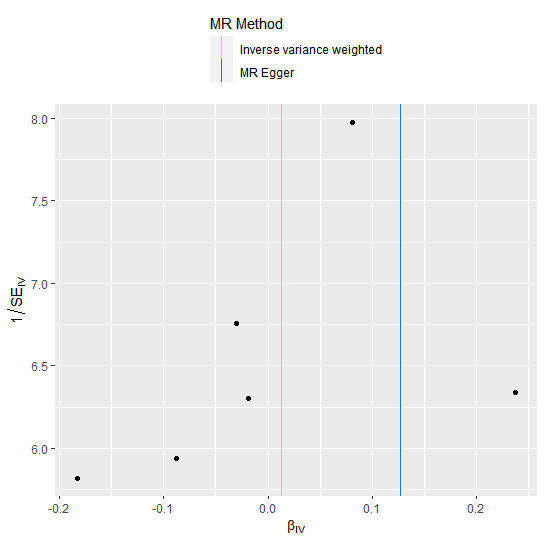

Supplement: Supplementary file 1 [file Data_Sheet_1.ZIP › Additional file 2 Funnel plot figure/S9 Stroke on KOA.png]

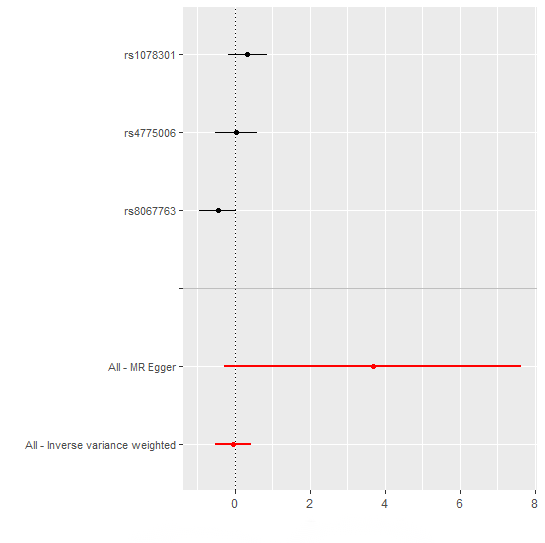

Supplement: Supplementary file 1 [file Data_Sheet_1.ZIP › Additional file 3 Forest plot figure/T1 KOA on CHD.png]

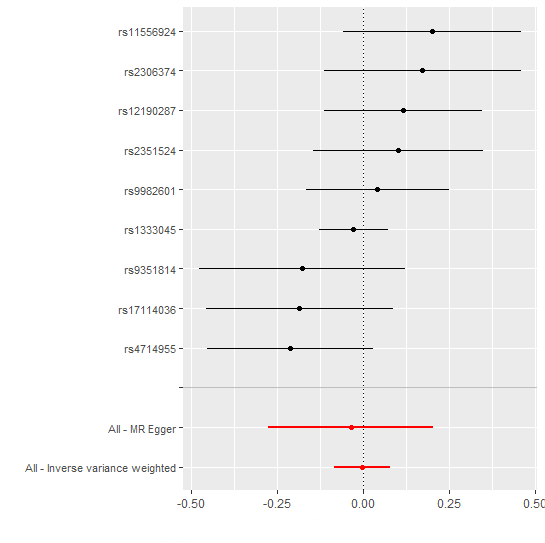

Supplement: Supplementary file 1 [file Data_Sheet_1.ZIP › Additional file 3 Forest plot figure/T10 CHD on HOA .png]

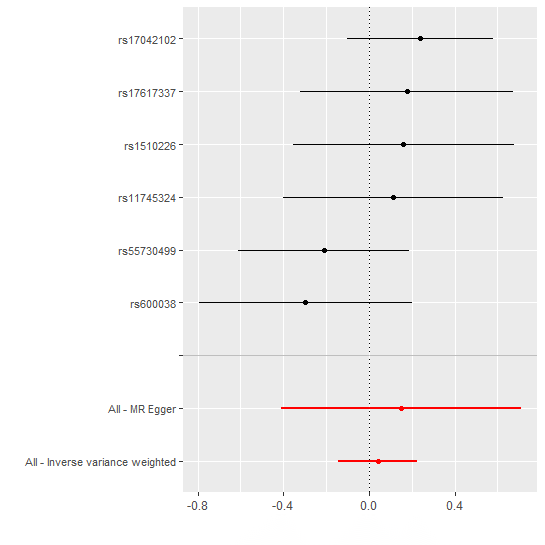

Supplement: Supplementary file 1 [file Data_Sheet_1.ZIP › Additional file 3 Forest plot figure/T11 HF on HOA.png]

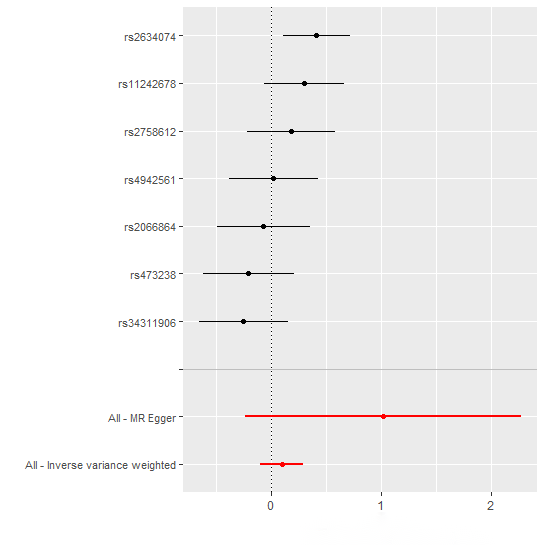

Supplement: Supplementary file 1 [file Data_Sheet_1.ZIP › Additional file 3 Forest plot figure/T12 Stroke on HOA.png]

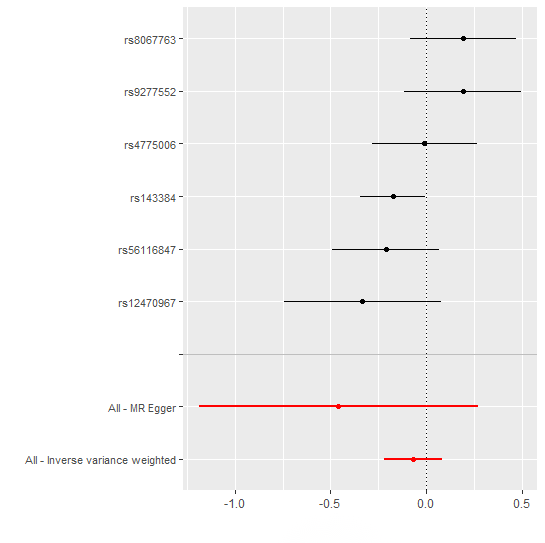

Supplement: Supplementary file 1 [file Data_Sheet_1.ZIP › Additional file 3 Forest plot figure/T2 KOA on HF.png]

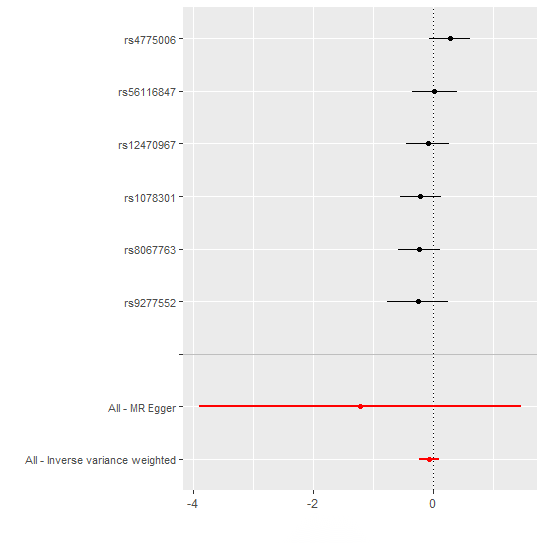

Supplement: Supplementary file 1 [file Data_Sheet_1.ZIP › Additional file 3 Forest plot figure/T3 KOA on Stroke.png]

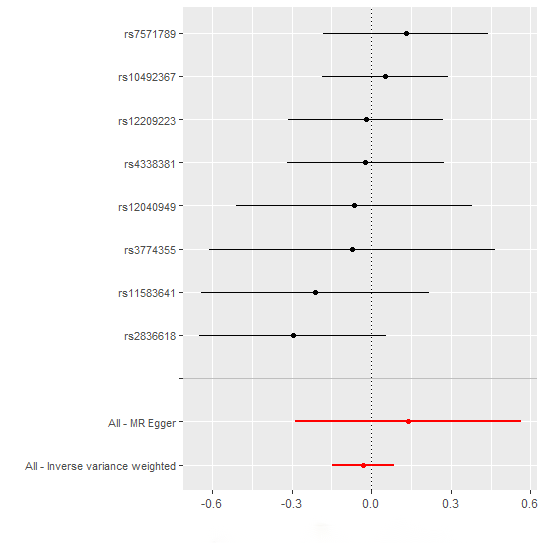

Supplement: Supplementary file 1 [file Data_Sheet_1.ZIP › Additional file 3 Forest plot figure/T4 HOA on CHD.png]

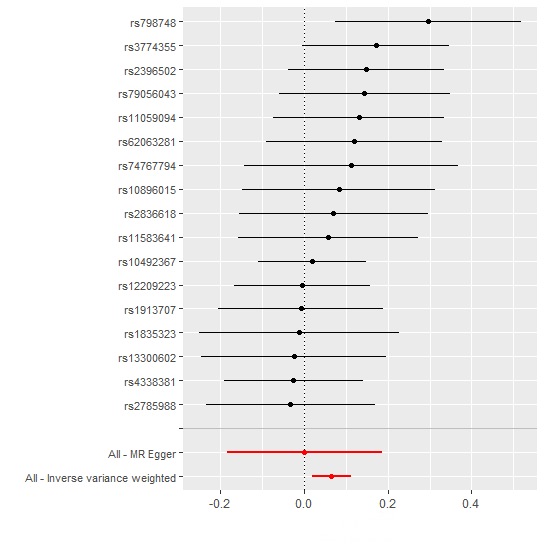

Supplement: Supplementary file 1 [file Data_Sheet_1.ZIP › Additional file 3 Forest plot figure/T5 HOA on HF.png]

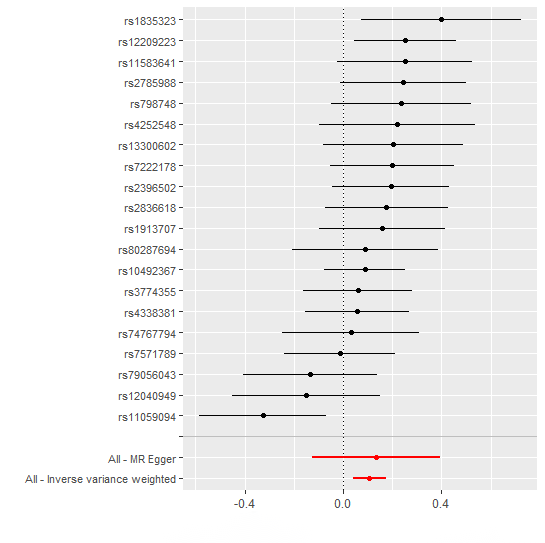

Supplement: Supplementary file 1 [file Data_Sheet_1.ZIP › Additional file 3 Forest plot figure/T6 HOA on Stroke.png]

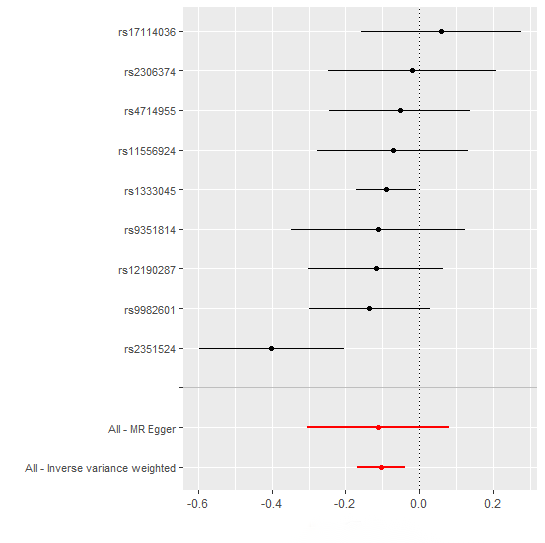

Supplement: Supplementary file 1 [file Data_Sheet_1.ZIP › Additional file 3 Forest plot figure/T7 CHD on KOA.png]

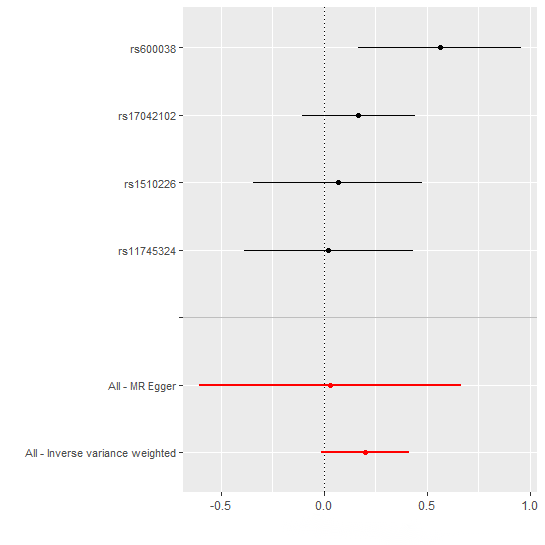

Supplement: Supplementary file 1 [file Data_Sheet_1.ZIP › Additional file 3 Forest plot figure/T8 HF on KOA.png]

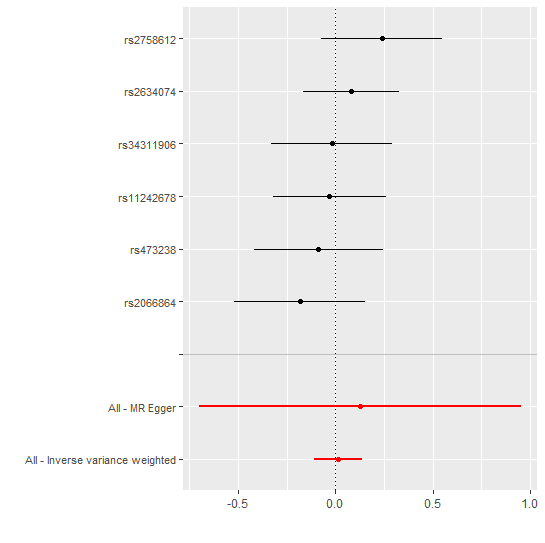

Supplement: Supplementary file 1 [file Data_Sheet_1.ZIP › Additional file 3 Forest plot figure/T9 Stroke on KOA.png]

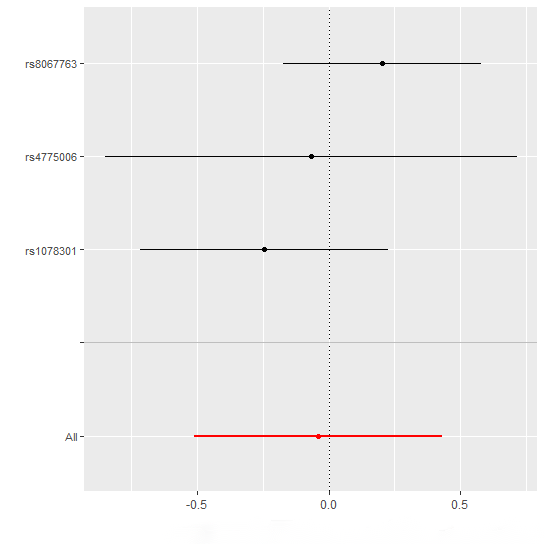

Supplement: Supplementary file 1 [file Data_Sheet_1.ZIP › Additional file 4 leave-one-out sensitivity analysis/H1 KOA on CHD.png]

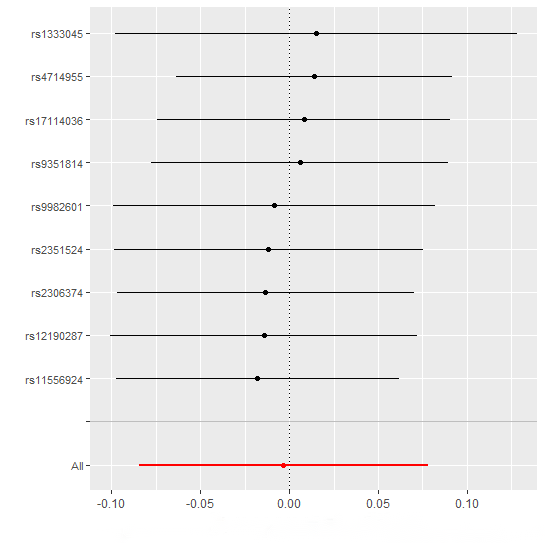

Supplement: Supplementary file 1 [file Data_Sheet_1.ZIP › Additional file 4 leave-one-out sensitivity analysis/H10 CHD on HOA.png]

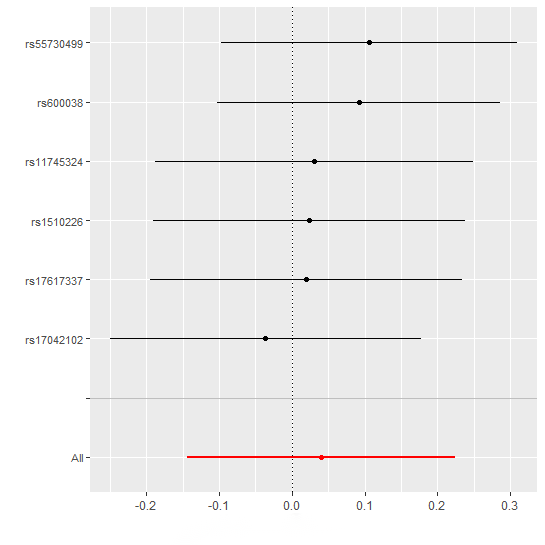

Supplement: Supplementary file 1 [file Data_Sheet_1.ZIP › Additional file 4 leave-one-out sensitivity analysis/H11 HF on HOA .png]

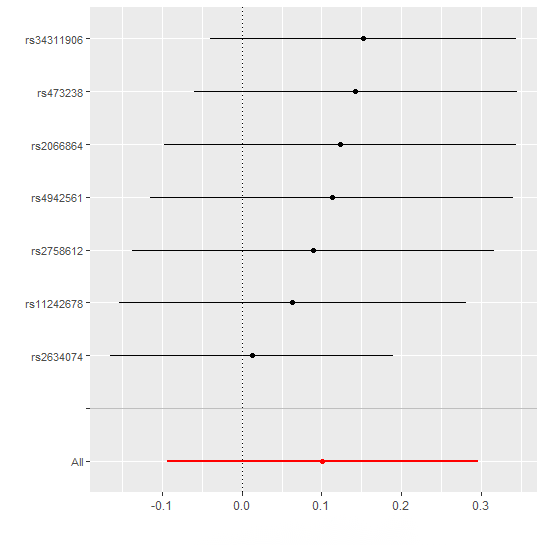

Supplement: Supplementary file 1 [file Data_Sheet_1.ZIP › Additional file 4 leave-one-out sensitivity analysis/H12 Stroke on HOA.png]

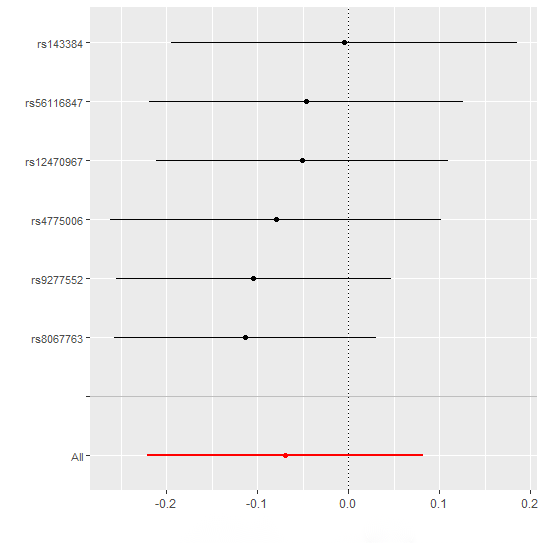

Supplement: Supplementary file 1 [file Data_Sheet_1.ZIP › Additional file 4 leave-one-out sensitivity analysis/H2 KOA on HF.png]

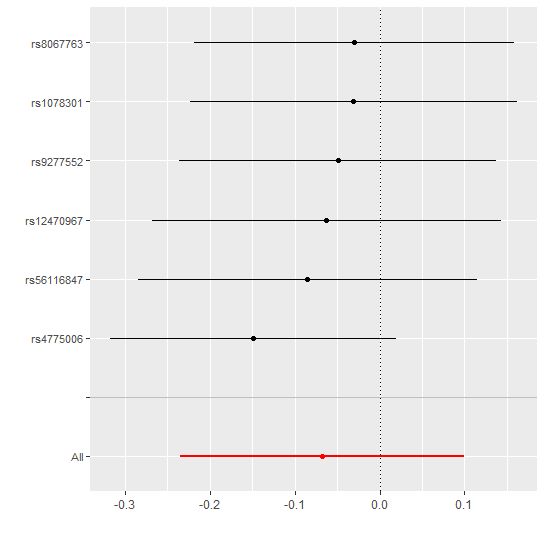

Supplement: Supplementary file 1 [file Data_Sheet_1.ZIP › Additional file 4 leave-one-out sensitivity analysis/H3 KOA on Stroke.png]

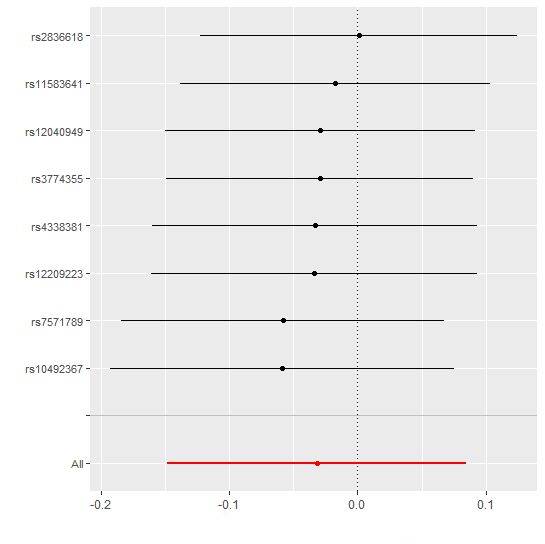

Supplement: Supplementary file 1 [file Data_Sheet_1.ZIP › Additional file 4 leave-one-out sensitivity analysis/H4 HOA on CHD.png]

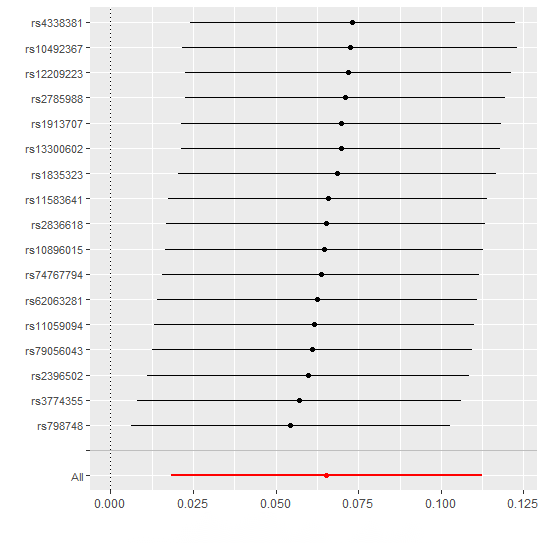

Supplement: Supplementary file 1 [file Data_Sheet_1.ZIP › Additional file 4 leave-one-out sensitivity analysis/H5 HOA on HF.png]

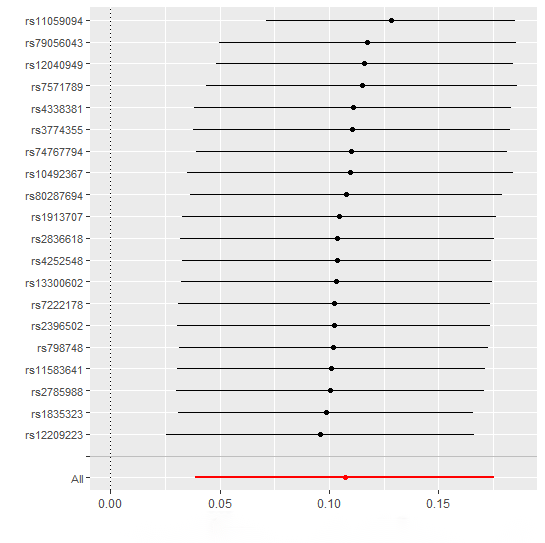

Supplement: Supplementary file 1 [file Data_Sheet_1.ZIP › Additional file 4 leave-one-out sensitivity analysis/H6 HOA to Stroke.png]

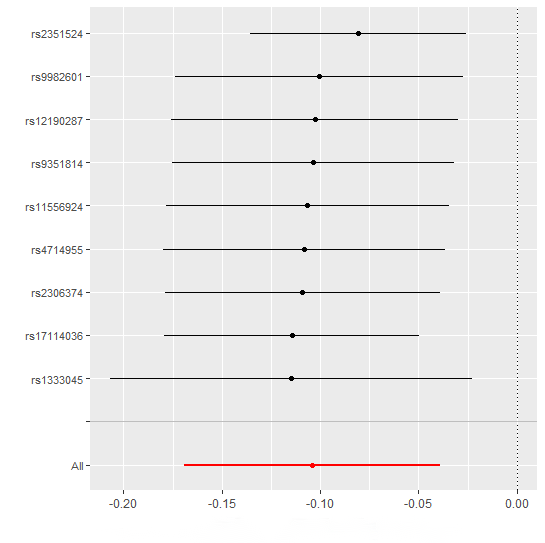

Supplement: Supplementary file 1 [file Data_Sheet_1.ZIP › Additional file 4 leave-one-out sensitivity analysis/H7 CHD on KOA.png]

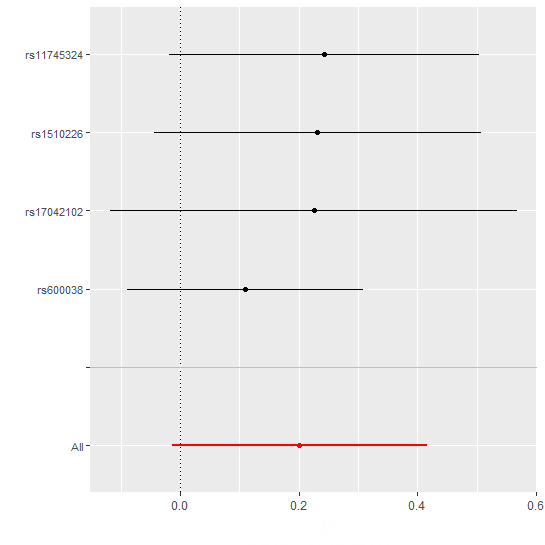

Supplement: Supplementary file 1 [file Data_Sheet_1.ZIP › Additional file 4 leave-one-out sensitivity analysis/H8 HF on KOA.png]

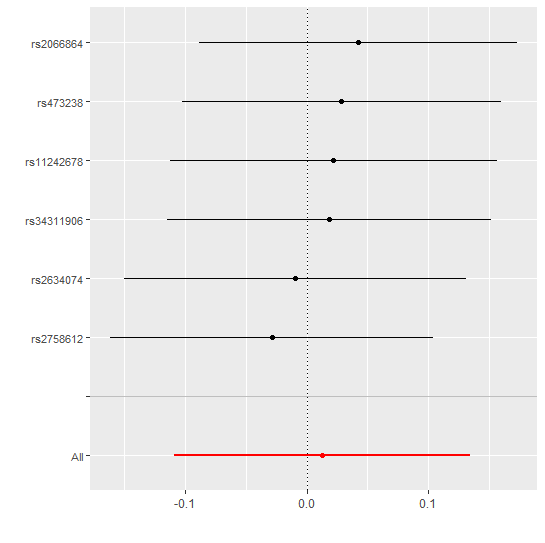

Supplement: Supplementary file 1 [file Data_Sheet_1.ZIP › Additional file 4 leave-one-out sensitivity analysis/H9 Stroke on KOA.png]
