# Supplementary material for: Osteoarthritis and cardiovascular disease: A Mendelian randomization study
Source: Front Cardiovasc Med. 2022 Nov 18;9:1025063. doi: 10.3389/fcvm.2022.1025063 (PMC9717609; doi:10.3389/fcvm.2022.1025063)
Supplement: Supplementary file 2 [file Data_Sheet_2.docx]

Supplementary Material

**Supplementary Table D1.** Association of the SNPs used as candidate genetic instruments from the GWAS for Mendelian randomization analyses of CHD and risk of KOA

**Supplementary Table D2.** Association of the SNPs used as candidate genetic instruments from the GWAS for Mendelian randomization analyses of CHD and risk of HOA

**Supplementary Table D3.** Association of the SNPs used as candidate genetic instruments from the GWAS for Mendelian randomization analyses of KOA and risk of CHD

**Supplementary Table D4.** Association of the SNPs used as candidate genetic instruments from the GWAS for Mendelian randomization analyses of HOA and risk of CHD

**Supplementary Table D5.** Association of the SNPs used as candidate genetic instruments from the GWAS for Mendelian randomization analyses of HF and risk of KOA

**Supplementary Table D6.** Association of the SNPs used as candidate genetic instruments from the GWAS for Mendelian randomization analyses of HF and risk of HOA

**Supplementary Table D7.** Association of the SNPs used as candidate genetic instruments from the GWAS for Mendelian randomization analyses of KOA and risk of HF

**Supplementary Table D8.** Association of the SNPs used as candidate genetic instruments from the GWAS for Mendelian randomization analyses of HOA and risk of HF

**Supplementary Table D9.** Association of the SNPs used as candidate genetic instruments from the GWAS for Mendelian randomization analyses of stroke and risk of KOA

**Supplementary Table D10.** Association of the SNPs used as candidate genetic instruments from the GWAS for Mendelian randomization analyses of stroke and risk of HOA

**Supplementary Table D11.** Association of the SNPs used as candidate genetic instruments from the GWAS for Mendelian randomization analyses of KOA and risk of stroke

**Supplementary Table D12.** Association of the SNPs used as candidate genetic instruments from the GWAS for Mendelian randomization analyses of HOA and risk of stroke

**Supplementary Table E1.** Abnormal SNPs in MR-PRESSO analysis

**Supplementary Table E2.** Abnormal SNPs in the one-and-leave method

**Supplementary Table F1.** MR-PRESSO test of all the results

**Supplementary Table H1.** The information about the source of the original data extracted from GWAS summary statistics

**Supplementary Table H2**. Confounder-related SNPs

| **Supplementary Table D1.** Association of the SNPs used as candidate genetic instruments from the GWAS for Mendelian randomization analyses of CHD and risk of KOA | | | | | | | | | | |
| --- | --- | --- | --- | --- | --- | --- | --- | --- | --- | --- |
| SNP | Chr | Pos | EA | OA | CHD (Exposure) | | | KOA (Outcome) | | |
|  |  |  |  |  | Beta | SE | *P*-value | Beta | SE | *P*-value |
| rs17114036 | 1 | 56962821 | G | A | -0.144965 | 0.0255686 | 1.43001E-08 | 0.0267 | 0.0201 | 0.1849 |
| rs2351524 | 2 | 203880992 | C | T | -0.13869 | 0.0206229 | 1.75995E-11 | -0.0142 | 0.0174 | 0.4159 |
| rs2306374 | 3 | 138119952 | C | T | 0.108439 | 0.019636 | 3.34003E-08 | 0.0186 | 0.0158 | 0.2382 |
| rs4714955 | 6 | 12903435 | T | C | -0.0997862 | 0.0145058 | 6.02976E-12 | 0.0211 | 0.0123 | 0.0874702 |
| rs9351814 | 6 | 72193707 | C | A | -0.0795946 | 0.0141883 | 2.01999E-08 | 0.0141 | 0.0121 | 0.2412 |
| rs12190287 | 6 | 134214525 | G | C | -0.103209 | 0.0156807 | 4.63981E-11 | -0.012 | 0.012 | 0.3193 |
| rs11556924 | 7 | 129663496 | T | C | -0.0905122 | 0.0151329 | 2.21998E-09 | -0.0182 | 0.0119 | 0.1278 |
| rs1333045 | 9 | 22119195 | C | T | 0.226084 | 0.019183 | 4.6302E-32 | -0.0062 | 0.0117 | 0.5933 |
| rs9982601 | 21 | 35599128 | T | C | 0.163991 | 0.0262564 | 4.21998E-10 | 0.0069 | 0.0173 | 0.6924 |
| Abbreviations: SNP,single-nucleotide polymorphism; Chr, chromosome; Pos, position; EA, effect allele; OA, other allele; SE, standard error; CHD, coronary heart disease; KOA, knee osteoarthritis. | | | | | | | | | | |

| **Supplementary Table D2.** Association of the SNPs used as candidate genetic instruments from the GWAS for Mendelian randomization analyses of CHD and risk of HOA | | | | | | | | | | |
| --- | --- | --- | --- | --- | --- | --- | --- | --- | --- | --- |
| SNP | Chr | Pos | EA | OA | CHD (Exposure) | | | HOA (Outcome) | | |
|  |  |  |  |  | Beta | SE | *P*-value | Beta | SE | *P*-value |
| rs17114036 | 1 | 56962821 | G | A | -0.144965 | 0.0255686 | 1.43001E-08 | 0.0267 | 0.0201 | 0.1849 |
| rs2351524 | 2 | 203880992 | C | T | -0.13869 | 0.0206229 | 1.75995E-11 | -0.0142 | 0.0174 | 0.4159 |
| rs2306374 | 3 | 138119952 | C | T | 0.108439 | 0.019636 | 3.34003E-08 | 0.0186 | 0.0158 | 0.2382 |
| rs4714955 | 6 | 12903435 | T | C | -0.0997862 | 0.0145058 | 6.02976E-12 | 0.0211 | 0.0123 | 0.0874702 |
| rs9351814 | 6 | 72193707 | C | A | -0.0795946 | 0.0141883 | 2.01999E-08 | 0.0141 | 0.0121 | 0.2412 |
| rs12190287 | 6 | 134214525 | G | C | -0.103209 | 0.0156807 | 4.63981E-11 | -0.012 | 0.012 | 0.3193 |
| rs11556924 | 7 | 129663496 | T | C | -0.0905122 | 0.0151329 | 2.21998E-09 | -0.0182 | 0.0119 | 0.1278 |
| rs1333045 | 9 | 22119195 | C | T | 0.226084 | 0.019183 | 4.6302E-32 | -0.0062 | 0.0117 | 0.5933 |
| rs9982601 | 21 | 35599128 | T | C | 0.163991 | 0.0262564 | 4.21998E-10 | 0.0069 | 0.0173 | 0.6924 |
| Abbreviations: SNP,single-nucleotide polymorphism; Chr, chromosome; Pos, position; EA, effect allele; OA, other allele; SE, standard error; CHD, coronary heart disease; HOA, hip osteoarthritis. | | | | | | | | | | |

| **Supplementary Table D3.** Association of the SNPs used as candidate genetic instruments from the GWAS for Mendelian randomization analyses of KOA and risk of CHD | | | | | | | | | | |
| --- | --- | --- | --- | --- | --- | --- | --- | --- | --- | --- |
| SNP | Chr | Pos | EA | OA | KOA (Exposure) | | | CHD (Outcome) | | |
|  |  |  |  |  | Beta | SE | *P*-value | Beta | SE | *P*-value |
| rs1078301 | 9 | 116909146 | T | A | 0.0679 | 0.0106 | 1.269E-10 | 0.023385 | 0.0175957 | 0.183842 |
| rs4775006 | 15 | 58215727 | A | C | 0.0578 | 0.0094 | 8.40001E-10 | 0.0017683 | 0.0165409 | 0.914866 |
| rs8067763 | 17 | 70012939 | A | G | -0.0566 | 0.0095 | 2.386E-09 | -0.0566 | 0.0141803 | 0.07027 |
| Abbreviations: SNP,single-nucleotide polymorphism; Chr, chromosome; Pos, position; EA, effect allele; OA, other allele; SE, standard error; CHD, coronary heart disease; KOA, knee osteoarthritis. | | | | | | | | | | |

| **Supplementary Table D4.** Association of the SNPs used as candidate genetic instruments from the GWAS for Mendelian randomization analyses of HOA and risk of CHD | | | | | | | | | | |
| --- | --- | --- | --- | --- | --- | --- | --- | --- | --- | --- |
| SNP | Chr | Pos | EA | OA | HOA (Exposure) | | | CHD(Outcome) | | |
|  |  |  |  |  | Beta | SE | *P*-value | Beta | SE | *P*-value |
| rs12040949 | 1 | 150447462 | T | C | -0.0665 | 0.012 | 2.83498E-08 | 0.0043528 | 0.0151101 | 0.773289 |
| rs11583641 | 1 | 183906245 | T | C | -0.0811 | 0.0131 | 5.57494E-10 | 0.0171182 | 0.0178181 | 0.336694 |
| rs4338381 | 1 | 103572927 | G | A | -0.095 | 0.0121 | 4.37119E-15 | 0.0021921 | 0.0143298 | 0.878417 |
| rs7571789 | 2 | 70714793 | C | T | -0.0886 | 0.0117 | 3.25987E-14 | -0.0114655 | 0.0139945 | 0.412624 |
| rs3774355 | 3 | 52817778 | A | G | 0.0907 | 0.0121 | 8.20163E-14 | -0.0066831 | 0.0249341 | 0.788675 |
| rs12209223 | 6 | 76164589 | A | C | 0.1558 | 0.0191 | 3.88329E-16 | -0.0032412 | 0.023188 | 0.888835 |
| rs10492367 | 12 | 28014970 | T | G | 0.1518 | 0.0148 | 1.24796E-24 | 0.0079109 | 0.0182848 | 0.665269 |
| rs2836618 | 21 | 40048295 | A | G | 0.0876 | 0.0132 | 3.20184E-11 | -0.0259742 | 0.01576 | 0.0993299 |
| Abbreviations: SNP,single-nucleotide polymorphism; Chr, chromosome; Pos, position; EA, effect allele; OA, other allele; SE, standard error; CHD, coronary heart disease; HOA, hip osteoarthritis. | | | | | | | | | | |

| **Supplementary Table D5.** Association of the SNPs used as candidate genetic instruments from the GWAS for Mendelian randomization analyses of HF and risk of KOA | | | | | | | | | | |
| --- | --- | --- | --- | --- | --- | --- | --- | --- | --- | --- |
| SNP | Chr | Pos | EA | OA | HF (Exposure) | | | KOA (Outcome) | | |
|  |  |  |  |  | Beta | SE | *P*-value | Beta | SE | *P*-value |
| rs17042102 | 4 | 111668626 | A | G | 0.1103 | 0.0121 | 5.70558E-20 | 0.0185 | 0.0154 | 0.2297 |
| rs11745324 | 5 | 137012171 | A | G | -0.0528 | 0.0095 | 2.34498E-08 | -0.0012 | 0.011 | 0.9109 |
| rs1510226 | 6 | 160816409 | C | T | 0.162 | 0.0285 | 1.26599E-08 | 0.011 | 0.0339 | 0.7461 |
| rs600038 | 9 | 136151806 | C | T | 0.0569 | 0.0096 | 3.67697E-09 | 0.032 | 0.0115 | 0.00520499 |
| Abbreviations: SNP,single-nucleotide polymorphism; Chr, chromosome; Pos, position; EA, effect allele; OA, other allele; SE, standard error; HF, heart failure; KOA, knee osteoarthritis. | | | | | | | | | | |

| **Supplementary Table D6.** Association of the SNPs used as candidate genetic instruments from the GWAS for Mendelian randomization analyses of HF and risk of HOA | | | | | | | | | | |
| --- | --- | --- | --- | --- | --- | --- | --- | --- | --- | --- |
| SNP | Chr | Pos | EA | OA | HF (Exposure) | | | HOA (Outcome) | | |
|  |  |  |  |  | Beta | SE | *P*-value | Beta | SE | *P*-value |
| rs17042102 | 4 | 111668626 | A | G | 0.1103 | 0.0121 | 5.70558E-20 | 0.0263 | 0.0193 | 0.1742 |
| rs11745324 | 5 | 137012171 | A | G | -0.0528 | 0.0095 | 2.34498E-08 | -0.0059 | 0.0138 | 0.667599 |
| rs1510226 | 6 | 160816409 | C | T | 0.162 | 0.0285 | 1.26599E-08 | 0.026 | 0.0427 | 0.5427 |
| rs55730499 | 6 | 161005610 | T | C | 0.1058 | 0.0157 | 1.83021E-11 | -0.0224 | 0.0215 | 0.2979 |
| rs600038 | 9 | 136151806 | C | T | 0.0569 | 0.0096 | 3.67697E-09 | -0.0169 | 0.0144 | 0.2406 |
| rs17617337 | 10 | 121426884 | T | C | -0.0561 | 0.0095 | 3.65401E-09 | -0.0099 | 0.0142 | 0.4851 |
| Abbreviations: SNP,single-nucleotide polymorphism; Chr, chromosome; Pos, position; EA, effect allele; OA, other allele; SE, standard error; HF, heart failure; HOA, hip osteoarthritis. | | | | | | | | | | |

| **Supplementary Table D8.** Association of the SNPs used as candidate genetic instruments from the GWAS for Mendelian randomization analyses of HOA and risk of HF | | | | | | | | | | |
| --- | --- | --- | --- | --- | --- | --- | --- | --- | --- | --- |
| SNP | Chr | Pos | EA | OA | HOA (Exposure) | | | HF (Outcome) | | |
|  |  |  |  |  | Beta | SE | *P*-value | Beta | SE | *P*-value |
| rs12040949 | 1 | 150447462 | T | C | -0.0665 | 0.012 | 2.83498E-08 | 0.0098 | 0.008 | 0.2216 |
| rs11583641 | 1 | 183906245 | T | C | -0.0811 | 0.0131 | 5.57494E-10 | -0.0047 | 0.0089 | 0.5956 |
| rs4338381 | 1 | 103572927 | G | A | -0.095 | 0.0121 | 4.37119E-15 | 0.0024 | 0.0081 | 0.763901 |
| rs74767794 | 1 | 184006128 | G | A | -0.0751 | 0.0126 | 2.556E-09 | -0.0085 | 0.0098 | 0.3853 |
| rs2785988 | 1 | 219744138 | A | C | 0.0828 | 0.0127 | 7.30466E-11 | -0.0027 | 0.0085 | 0.751 |
| rs7571789 | 2 | 70714793 | C | T | -0.0886 | 0.0117 | 3.25987E-14 | 0.0093 | 0.0078 | 0.2332 |
| rs1835323 | 2 | 43512130 | T | C | -0.0673 | 0.0123 | 4.55795E-08 | 0.0008 | 0.0082 | 0.9209 |
| rs3774355 | 3 | 52817778 | A | G | 0.0907 | 0.0121 | 8.20163E-14 | 0.0155 | 0.0081 | 0.0557905 |
| rs798748 | 4 | 1716770 | C | T | 0.0715 | 0.012 | 2.49799E-09 | 0.0212 | 0.0081 | 0.00910899 |
| rs1913707 | 4 | 13039440 | G | A | -0.0795 | 0.012 | 2.9621E-11 | 0.0006 | 0.008 | 0.9414 |
| rs12209223 | 6 | 76164589 | A | C | 0.1558 | 0.0191 | 3.88329E-16 | -0.0009 | 0.0129 | 0.9442 |
| rs2396502 | 6 | 45357699 | C | A | 0.0842 | 0.012 | 2.11787E-12 | 0.0124 | 0.008 | 0.1201 |
| rs80287694 | 6 | 55636940 | G | A | 0.1093 | 0.0184 | 2.65999E-09 | -0.0101 | 0.013 | 0.436 |
| rs13300602 | 9 | 129412938 | G | A | 0.0716 | 0.0119 | 1.65402E-09 | -0.0018 | 0.0081 | 0.8235 |
| rs10896015 | 11 | 65323725 | A | G | -0.0782 | 0.0132 | 2.73502E-09 | -0.0065 | 0.0092 | 0.4789 |
| rs10492367 | 12 | 28014970 | T | G | 0.1518 | 0.0148 | 1.24796E-24 | 0.003 | 0.01 | 0.7663 |
| rs79056043 | 12 | 59289598 | G | A | 0.1625 | 0.0268 | 1.32999E-09 | 0.0234 | 0.0169 | 0.1677 |
| rs11059094 | 12 | 122606837 | T | C | 0.0759 | 0.0117 | 7.37734E-11 | 0.0099 | 0.0079 | 0.2091 |
| rs62063281 | 17 | 44038785 | G | A | 0.0964 | 0.014 | 5.29785E-12 | 0.0115 | 0.0103 | 0.2621 |
| rs2836618 | 21 | 40048295 | A | G | 0.0876 | 0.0132 | 3.20184E-11 | 0.0061 | 0.0101 | 0.549 |
| Abbreviations: SNP,single-nucleotide polymorphism; Chr, chromosome; Pos, position; EA, effect allele; OA, other allele; SE, standard error; HF, heart failure; HOA, hip osteoarthritis. | | | | | | | | | | |

| **Supplementary Table D7.** Association of the SNPs used as candidate genetic instruments from the GWAS for Mendelian randomization analyses of KOA and risk of HF | | | | | | | | | | |
| --- | --- | --- | --- | --- | --- | --- | --- | --- | --- | --- |
| SNP | Chr | Pos | EA | OA | KOA (Exposure) | | | HF (Outcome) | | |
|  |  |  |  |  | Beta | SE | *P*-value | Beta | SE | *P*-value |
| rs12470967 | 2 | 192671981 | G | A | -0.0584 | 0.0103 | 1.49799E-08 | 0.0194 | 0.0122 | 0.1121 |
| rs9277552 | 6 | 33055501 | T | C | -0.064 | 0.0114 | 1.96902E-08 | -0.0122 | 0.01 | 0.2221 |
| rs56116847 | 12 | 123835233 | A | G | 0.0612 | 0.0097 | 3.19396E-10 | -0.0129 | 0.0087 | 0.1382 |
| rs4775006 | 15 | 58215727 | A | C | 0.0578 | 0.0094 | 8.40001E-10 | -0.0005 | 0.0081 | 0.9529 |
| rs8067763 | 17 | 70012939 | A | G | -0.0566 | 0.0095 | 2.386E-09 | -0.0109 | 0.008 | 0.1698 |
| rs143384 | 20 | 34025756 | G | A | -0.0935 | 0.0095 | 4.77309E-23 | 0.0162 | 0.0081 | 0.0474297 |
| Abbreviations: SNP,single-nucleotide polymorphism; Chr, chromosome; Pos, position; EA, effect allele; OA, other allele; SE, standard error; HF, heart failure; KOA, knee osteoarthritis. | | | | | | | | | | |

| **Supplementary Table D9.** Association of the SNPs used as candidate genetic instruments from the GWAS for Mendelian randomization analyses of stroke and risk of KOA | | | | | | | | | | |
| --- | --- | --- | --- | --- | --- | --- | --- | --- | --- | --- |
| SNP | Chr | Pos | EA | OA | stroke (Exposure) | | | KOA (Outcome) | | |
|  |  |  |  |  | Beta | SE | *P*-value | Beta | SE | *P*-value |
| rs2758612 | 1 | 156205301 | C | T | -0.0653 | 0.0111 | 3.67697E-09 | -0.0155 | 0.0103 | 0.1324 |
| rs34311906 | 4 | 113732090 | C | T | 0.0649 | 0.0113 | 1.06601E-08 | -0.0012 | 0.0103 | 0.9051 |
| rs2634074 | 4 | 111677041 | A | T | -0.0941 | 0.0121 | 5.90473E-15 | -0.0076 | 0.0118 | 0.5188 |
| rs2066864 | 4 | 155525695 | A | G | 0.0634 | 0.0115 | 3.51399E-08 | -0.0116 | 0.0109 | 0.2846 |
| rs11242678 | 6 | 1337180 | T | C | 0.0723 | 0.0114 | 2.70302E-10 | -0.0022 | 0.0107 | 0.8336 |
| rs473238 | 11 | 102700360 | C | T | -0.0831 | 0.0147 | 1.65101E-08 | 0.0073 | 0.014 | 0.599799 |
| Abbreviations: SNP,single-nucleotide polymorphism; Chr, chromosome; Pos, position; EA, effect allele; OA, other allele; SE, standard error; KOA, knee osteoarthritis. | | | | | | | | | | |

| **Supplementary Table D10.** Association of the SNPs used as candidate genetic instruments from the GWAS for Mendelian randomization analyses of stroke and risk of HOA | | | | | | | | | | |
| --- | --- | --- | --- | --- | --- | --- | --- | --- | --- | --- |
| SNP | Chr | Pos | EA | OA | stroke (Exposure) | | | HOA (Outcome) | | |
|  |  |  |  |  | Beta | SE | *P*-value | Beta | SE | *P*-value |
| rs34311906 | 4 | 113732090 | C | T | 0.0649 | 0.0113 | 1.06601E-08 | -0.0161 | 0.0133 | 0.2251 |
| rs2066864 | 4 | 155525695 | A | G | 0.0634 | 0.0115 | 3.51399E-08 | -0.0042 | 0.0136 | 0.7597 |
| rs473238 | 11 | 102700360 | C | T | -0.0831 | 0.0147 | 1.65101E-08 | 0.0168 | 0.0176 | 0.3383 |
| rs4942561 | 13 | 47209347 | T | G | 0.0655 | 0.0116 | 1.77101E-08 | 0.0014 | 0.0135 | 0.9184 |
| Abbreviations: SNP,single-nucleotide polymorphism; Chr, chromosome; Pos, position; EA, effect allele; OA, other allele; SE, standard error; HOA, hip osteoarthritis. | | | | | | | | | | |

| **Supplementary Table D11.** Association of the SNPs used as candidate genetic instruments from the GWAS for Mendelian randomization analyses of KOA and risk of stroke | | | | | | | | | | |
| --- | --- | --- | --- | --- | --- | --- | --- | --- | --- | --- |
| SNP | Chr | Pos | EA | OA | KOA (Exposure) | | | stroke (Outcome) | | |
|  |  |  |  |  | Beta | SE | *P*-value | Beta | SE | *P*-value |
| rs12470967 | 2 | 192671981 | G | A | -0.0584 | 0.0103 | 1.49799E-08 | 0.0053 | 0.0108 | 0.6237 |
| rs9277552 | 6 | 33055501 | T | C | -0.064 | 0.0114 | 1.96902E-08 | 0.0162 | 0.0166 | 0.3267 |
| rs1078301 | 9 | 116909146 | T | A | 0.0679 | 0.0106 | 1.269E-10 | -0.0147 | 0.0118 | 0.2134 |
| rs56116847 | 12 | 123835233 | A | G | 0.0612 | 0.0097 | 3.19396E-10 | 0.0014 | 0.0117 | 0.9047 |
| rs4775006 | 15 | 58215727 | A | C | 0.0578 | 0.0094 | 8.40001E-10 | 0.0159 | 0.0102 | 0.1194 |
| rs8067763 | 17 | 70012939 | A | G | -0.0566 | 0.0095 | 2.386E-09 | 0.0132 | 0.0102 | 0.1956 |
| Abbreviations: SNP,single-nucleotide polymorphism; Chr, chromosome; Pos, position; EA, effect allele; OA, other allele; SE, standard error; KOA, knee osteoarthritis. | | | | | | | | | | |

| **Supplementary Table D12.** Association of the SNPs used as candidate genetic instruments from the GWAS for Mendelian randomization analyses of HOA and risk of stroke | | | | | | | | | | |
| --- | --- | --- | --- | --- | --- | --- | --- | --- | --- | --- |
| SNP | Chr | Pos | EA | OA | HOA (Exposure) | | | stroke(Outcome) | | |
|  |  |  |  |  | Beta | SE | *P*-value | Beta | SE | *P*-value |
| rs12040949 | 1 | 150447462 | T | C | -0.0665 | 0.012 | 2.83498E-08 | 0.0102 | 0.0102 | 0.3306 |
| rs11583641 | 1 | 183906245 | T | C | -0.0811 | 0.0131 | 5.57494E-10 | -0.0203 | 0.0113 | 0.07193 |
| rs4338381 | 1 | 103572927 | G | A | -0.095 | 0.0121 | 4.37119E-15 | -0.0054 | 0.0102 | 0.5988 |
| rs74767794 | 1 | 184006128 | G | A | -0.0751 | 0.0126 | 2.556E-09 | -0.0023 | 0.0107 | 0.8313 |
| rs2785988 | 1 | 219744138 | A | C | 0.0828 | 0.0127 | 7.30466E-11 | 0.0201 | 0.0108 | 0.0632601 |
| rs7571789 | 2 | 70714793 | C | T | -0.0886 | 0.0117 | 3.25987E-14 | 0.0012 | 0.0102 | 0.9034 |
| rs1835323 | 2 | 43512130 | T | C | -0.0673 | 0.0123 | 4.55795E-08 | -0.0268 | 0.0112 | 0.0166399 |
| rs3774355 | 3 | 52817778 | A | G | 0.0907 | 0.0121 | 8.20163E-14 | 0.0054 | 0.0102 | 0.595899 |
| rs798748 | 4 | 1716770 | C | T | 0.0715 | 0.012 | 2.49799E-09 | 0.0169 | 0.0104 | 0.1054 |
| rs1913707 | 4 | 13039440 | G | A | -0.0795 | 0.012 | 2.9621E-11 | -0.0127 | 0.0104 | 0.2226 |
| rs12209223 | 6 | 76164589 | A | C | 0.1558 | 0.0191 | 3.88329E-16 | 0.0394 | 0.0165 | 0.0167799 |
| rs2396502 | 6 | 45357699 | C | A | 0.0842 | 0.012 | 2.11787E-12 | 0.0164 | 0.0102 | 0.1062 |
| rs80287694 | 6 | 55636940 | G | A | 0.1093 | 0.0184 | 2.65999E-09 | 0.0099 | 0.0166 | 0.551899 |
| rs13300602 | 9 | 129412938 | G | A | 0.0716 | 0.0119 | 1.65402E-09 | 0.0146 | 0.0104 | 0.1584 |
| rs10492367 | 12 | 28014970 | T | G | 0.1518 | 0.0148 | 1.24796E-24 | 0.0134 | 0.0127 | 0.2916 |
| rs79056043 | 12 | 59289598 | G | A | 0.1625 | 0.0268 | 1.32999E-09 | -0.0219 | 0.0226 | 0.3333 |
| rs11059094 | 12 | 122606837 | T | C | 0.0759 | 0.0117 | 7.37734E-11 | -0.0248 | 0.01 | 0.0129199 |
| rs7222178 | 17 | 59652282 | A | T | 0.0965 | 0.0146 | 3.77485E-11 | 0.0194 | 0.0124 | 0.118 |
| rs4252548 | 19 | 55879672 | T | C | 0.2785 | 0.0396 | 1.95614E-12 | 0.0612 | 0.0449 | 0.1728 |
| rs2836618 | 21 | 40048295 | A | G | 0.0876 | 0.0132 | 3.20184E-11 | 0.0154 | 0.0112 | 0.1688 |
| Abbreviations: SNP,single-nucleotide polymorphism; Chr, chromosome; Pos, position; EA, effect allele; OA, other allele; SE, standard error; HOA, hip osteoarthritis. | | | | | | | | | | |

| **Supplementary Table E1.** Abnormal SNPs in MR-PRESSO analysis | | | | | |
| --- | --- | --- | --- | --- | --- |
| Exposures | Outcomes | Deleted SNP | beta | SE | P-value |
| stroke | KOA | rs4942561 | 0.0655 | 0.0116 | 1.77101E-08 |
| HOA | stroke | rs10896015 | -0.0782 | 0.0132 | 2.73502E-09 |
| HOA | stroke | rs11059094 | 0.0759 | 0.0117 | 7.37734E-11 |
| Abbreviations: SNP,single-nucleotide polymorphism; SE, standard error; HOA, hip osteoarthritis; KOA, knee osteoarthritis | | | | | |

| **Supplementary Table E2.** Abnormal SNPs in the one-and-leave method | | | | | |
| --- | --- | --- | --- | --- | --- |
| Exposures | Outcomes | Deleted SNP | beta | SE | P-value |
| HOA | HF | rs12040949 | -0.0665 | 0.012 | 2.83498E-08 |
| HOA | HF | rs7571789 | -0.0886 | 0.0117 | 3.25987E-14 |
| HOA | HF | rs80287694 | 0.1093 | 0.0184 | 2.65999E-09 |
| HF | KOA | rs55730499 | 0.1058 | 0.0157 | 1.83021E-11 |
| HF | KOA | rs17617337 | -0.0561 | 0.0095 | 3.65401E-09 |
| Abbreviations: SNP,single-nucleotide polymorphism; SE, standard error; HF, heart failure;HOA, hip osteoarthritis; KOA, knee osteoarthritis | | | | | |

| **Supplementary Table F1.** MR-PRESSO test of all the results | | | | |
| --- | --- | --- | --- | --- |
| Exposure | Outcomes | Global p-value | Number of outliers | Distortion p-value |
| CHD | KOA | 0.230 | 0 | NA |
| CHD | HOA | 0.213 | 0 | NA |
| KOA | CHD | 0.147 | 0 | NA |
| HOA | CHD | 0.775 | 0 | NA |
| HF | KOA | 0.076 | 0 | NA |
| HF | HOA | 0.401 | 0 | NA |
| KOA | HF | 0.118 | 0 | NA |
| HOA | HF | 0.712 | 0 | NA |
| stroke | KOA | 0.575 | 0 | NA |
| stroke | HOA | 0.102 | 0 | NA |
| KOA | stroke | 0.293 | 0 | NA |
| HOA | stroke | 0.513 | 0 | NA |
| Abbreviations: SNP,single-nucleotide polymorphism; CHD,coronary heart disease; HF, heart failure;HOA, hip osteoarthritis; KOA, knee osteoarthritis; MR-PRESSO, mendelian Randomization Pleiotropy RESidual Sum and Outlier | | | | |

| **Supplementary Table H1** The information about the source of the original data extracted from GWAS summary statistics | | | | |
| --- | --- | --- | --- | --- |
| Trait | Sample size | Number of SNPs | populations | First author |
| CHD | 86,995 | 2,415,020 | European | Schunkert H, et al |
| HF | 977,323 | 7,773,021 | European | Malik R, et al |
| stroke | 440,328 | 8,296,492 | European | Shah S, et al |
| KOA | 403,124 | 29,999,696 | European | Tachmazidou I, et al |
| HOA | 393,873 | 29,771,219 | European | Tachmazidou I, et al |
| Abbreviations: CHD, coronary heart disease; HF, heart failure; KOA, knee osteoarthritis; HOA, hip osteoarthritis | | | | |

| **Supplementary Table H2.** Confounder-related SNPs | | | | | |
| --- | --- | --- | --- | --- | --- |
| Exposures | Outcomes | Deleted SNP | SE | beta | P-value |
| CHD | KOA | rs599839 | 0.0169259 | 0.106715 | 2.89001E-10 |
| CHD | KOA | rs7651039 | 0.0252631 | 0.142119 | 1.84999E-08 |
| CHD | KOA | rs10455872 | 0.038112 | 0.27787 | 3.08035E-13 |
| CHD | KOA | rs964184 | 0.0204991 | -0.12596 | 8.01992E-10 |
| CHD | KOA | rs2219939 | 0.016288 | -0.0990217 | 1.21001E-09 |
| CHD | KOA | rs1122608 | 0.0208429 | -0.127428 | 9.72994E-10 |
| CHD | HOA | rs599839 | 0.0169259 | 0.106715 | 2.89001E-10 |
| CHD | HOA | rs7651039 | 0.0252631 | 0.142119 | 1.84999E-08 |
| CHD | HOA | rs10455872 | 0.038112 | 0.27787 | 3.08035E-13 |
| CHD | HOA | rs964184 | 0.0204991 | -0.12596 | 8.01992E-10 |
| CHD | HOA | rs2219939 | 0.016288 | -0.0990217 | 1.21001E-09 |
| CHD | HOA | rs1122608 | 0.0208429 | -0.127428 | 9.72994E-10 |
| HF | KOA | rs4135240 | 0.0084 | 6.83801E-09 | -0.0486 |
| HF | KOA | rs660240 | 0.0097 | 0.0611 | 3.25102E-10 |
| HF | KOA | rs56094641 | 0.008 | 0.0454 | 1.20801E-08 |
| HF | HOA | rs4135240 | 0.0084 | 6.83801E-09 | -0.0486 |
| HF | HOA | rs660240 | 0.0097 | 0.0611 | 3.25102E-10 |
| HF | HOA | rs56094641 | 0.008 | 0.0454 | 1.20801E-08 |
| HOA | HF | rs115740542 | 0.0224 | 0.1263 | 1.60398E-08 |
| HOA | HF | rs4252548 | 0.0396 | 0.2785 | 1.95614E-12 |
| Stroke | HOA | rs3184504 | 0.0101 | -0.0779 | 1.22914E-14 |
| Stroke | HOA | rs2107595 | 0.0132 | 0.0882 | 2.32809E-11 |
| Stroke | KOA | rs2107595 | 0.0132 | 0.0882 | 2.32809E-11 |
| Stroke | KOA | rs3184504 | 0.0101 | -0.0779 | 1.22914E-14 |
| HOA | Stroke | rs115740542 | 0.0224 | 0.1263 | 1.60398E-08 |
| HOA | Stroke | rs62063281 | 0.014 | 0.0964 | 5.29785E-12 |
| Abbreviations: SNP,single-nucleotide polymorphism; SE, standard error; HF, heart failure;HOA, hip osteoarthritis; KOA, knee osteoarthritis; CHD, coronary heart disease. | | | | | |
